# Epigenetic regulatory networks in the pathogenesis and treatment of osteoporosis subtypes

**DOI:** 10.1186/s43556-026-00630-1

**Published:** 2026-10-21

**Authors:** Zhengda Ren, Bingyang Dai, Dequan Chen, Zehui Li, Keyu Meng, Shanwei Lan, Canzhao Liu, Zhongmin Zhang, Xiaoyi Mo

**Affiliations:** 1 Division of Spine Surgery, Department of Orthopaedics, Nanfang Hospital, Southern Medical University, Guangzhou, 510515 China; 2 Department of Biomedical Engineering, The Hong Kong Polytechnic University, Hong Kong, 999077 China; 3 Department of Cardiology, Laboratory of Heart Center, Translational Medicine Research Center, Zhujiang Hospital, Southern Medical University, Guangzhou, Guangdong 510282 China; 4 Department of Joint and Orthopedics, Zhujiang Hospital, Southern Medical University, Guangzhou, Guangdong 510282 China

**Keywords:** Osteoporosis, Epigenetics, Bone remodeling, Osteoblasts, Osteoclasts

## Abstract

Osteoporosis is a systemic skeletal disorder characterized by reduced bone mineral density and heightened bone fragility, encompassing primary (senile and postmenopausal) and secondary (glucocorticoid-induced and diabetic) subtypes. Maintaining skeletal health relies on the dynamic balance between osteoblast-mediated bone formation and osteoclast-mediated bone resorption. Among the multitude of factors orchestrating this bone remodeling process, epigenetics, which regulates gene expression via reversible chemical modifications without altering the primary DNA sequence, plays an essential role in maintaining bone homeostasis. While previous research has identified numerous epigenetic factors in bone metabolism, a comprehensive synthesis of their specific contributions to different osteoporosis subtypes and the corresponding therapeutic strategies remains lacking. This review outlines the major epigenetic mechanisms involved in bone metabolism, including DNA methylation, histone modifications, RNA modifications, and non-coding RNAs. Subsequently, we comprehensively elucidate how the dysregulation of these epigenetic landscapes drives the pathogenesis of distinct osteoporosis subtypes, emphasizing their roles in disrupting the osteoblast-osteoclast axis. Furthermore, we review emerging epigenetic-based therapeutics across distinct osteoporosis subtypes, ranging from systemic pharmacological inhibitors, such as DNA methyltransferase and histone demethylase inhibitors, to advanced delivery platforms targeting the skeletal microenvironment, such as engineered extracellular vesicles and functionalized nanocarriers. By integrating molecular mechanistic insights with these diverse therapeutic strategies, this review establishes a comprehensive framework for understanding the epigenetic heterogeneity in the pathogenesis and therapeutics of osteoporosis. Ultimately, it provides essential directions for future investigations, offering innovative epigenetic strategies to tackle various osteoporosis subtypes.

## Introduction

Osteoporosis is a systemic skeletal disease characterized by reduced bone mass and deterioration of bone microstructure [1]. As a major global public health concern, low bone mineral density and osteoporosis impose a massive clinical and economic burden worldwide, predominantly affecting postmenopausal women and the ageing population. Recent Global Burden of Disease estimates indicate that skeletal fragility attributable to low bone mineral density contributes to over 17 million disability-adjusted life-years and 477,000 deaths each year, with absolute fracture-related deaths increasing by 127.1% over the past three decades [2]. Normal bone metabolism depends on the dynamic balance between osteoblast-mediated bone formation and osteoclast-mediated bone resorption [3]. This process is regulated by multiple factors, including genetics [4], hormones [5], cytokines [6, 7], and mechanical stress [3]. Osteoporosis is generally classified into primary and secondary forms. Primary osteoporosis includes senile osteoporosis, postmenopausal osteoporosis (PMOP), and idiopathic osteoporosis [8]. Secondary osteoporosis can arise from underlying diseases, medications, or nutritional deficiencies, such as diabetes-related and glucocorticoid-induced osteoporosis [9].

Among the multitude of factors orchestrating the dynamic balance of the bone remodeling process, epigenetic regulation has emerged as a fundamental driver [10]. Epigenetics is defined as reversible changes in gene expression that modulate cellular phenotype and function without altering the primary DNA sequence. The principal epigenetic mechanisms encompass DNA methylation, histone modification, mRNA modification and regulation mediated by non-coding RNAs [11]. Notably, different types of osteoporosis exhibit distinct features at the level of epigenetic regulation. Specifically, senile osteoporosis is mainly characterized by age-associated epigenetic drift, including global DNA hypomethylation, dysregulated histone marks, and senescence-related non-coding RNA profiles, which impair mesenchymal stem cell osteogenic potential and bone formation [12]. In contrast, in PMOP, estrogen deficiency predominantly reshapes the epigenetic landscape by altering DNA methylation patterns and histone modifications in osteoclast- and osteoblast-related genes, thereby promoting excessive bone resorption [13]. Moreover, glucocorticoid-induced osteoporosis (GIOP) involves epigenetic repression of osteogenic genes and activation of adipogenic pathways through histone deacetylation and microRNA regulation [14]. Overall, aberrant epigenetic regulation disrupts the dynamic balance between bone formation and bone resorption, thereby driving the onset and progression of different types of osteoporosis.

The dynamic and reversible nature of epigenetic modifications renders them promising targets for restoring bone homeostasis. Recent advancements have identified a diverse array of epigenetic-based therapeutics tailored to distinct osteoporosis subtypes. These interventions include systemic pharmacological agents and targeted therapies directed at the skeletal microenvironment. On the one hand, systemic interventions, such as broad-spectrum DNA methyltransferase and histone-modifying enzyme inhibitors, have demonstrated efficacy in modulating epigenetic networks to rescue osteogenesis and ameliorate bone loss [15, 16]. On the other hand, to concentrate therapeutic effects within the bone niche and thereby avoid off-target effects, the field is advancing toward tissue-specific delivery platforms. Specifically, engineered extracellular vesicles, lipid nanoparticles, and viral vectors are now being deployed to deliver epigenetic modulators into the skeletal microenvironment [17–19]. Together, these diverse strategies, from systemic regulation to local targeted delivery, provide epigenetic therapeutic approaches for rebalancing the osteoblast-osteoclast axis, and highlight a promising translational avenue for osteoporosis treatment.

Despite these advances, a synthesis integrating both the epigenetic pathogenesis and emerging therapeutics across distinct osteoporosis subtypes remains lacking. Therefore, this review aims to provide a comprehensive overview of the regulatory networks associated with osteoporosis from an epigenetic perspective. First, we outline the four major epigenetic modifications and their dynamic regulatory mechanisms in bone metabolism. Subsequently, we elucidate how the dysregulation of these epigenetic modifications drives the pathogenesis of distinct osteoporosis subtypes, emphasizing their roles in disrupting the osteoblast-osteoclast axis. Finally, we summarize epigenetic-based therapeutics tailored to distinct osteoporosis subtypes, including systemic regulation and precision targeted delivery. By establishing a holistic theoretical framework, this review offers translational perspectives to guide future research and precision interventions for osteoporosis.

## Overview of epigenetics

Epigenetics refers to the mechanism of regulating gene expression patterns through reversible chemical modifications without changing the DNA sequence [20]. Epigenetic regulation is dynamic, reversible and environmentally responsive, making it an important bridge between genetic factors and the external environment, such as nutrition, hormones and inflammation [21, 22]. These modifications can affect chromatin structure, DNA accessibility and transcription factor binding ability, thus determining cell fate, tissue function and the occurrence and development of diseases [23, 24]. Epigenetic modifications mainly include the following four mechanisms: DNA methylation, histone modification, mRNA modification and non-coding RNA [11].

### DNA methylation

**Fig. 1:**
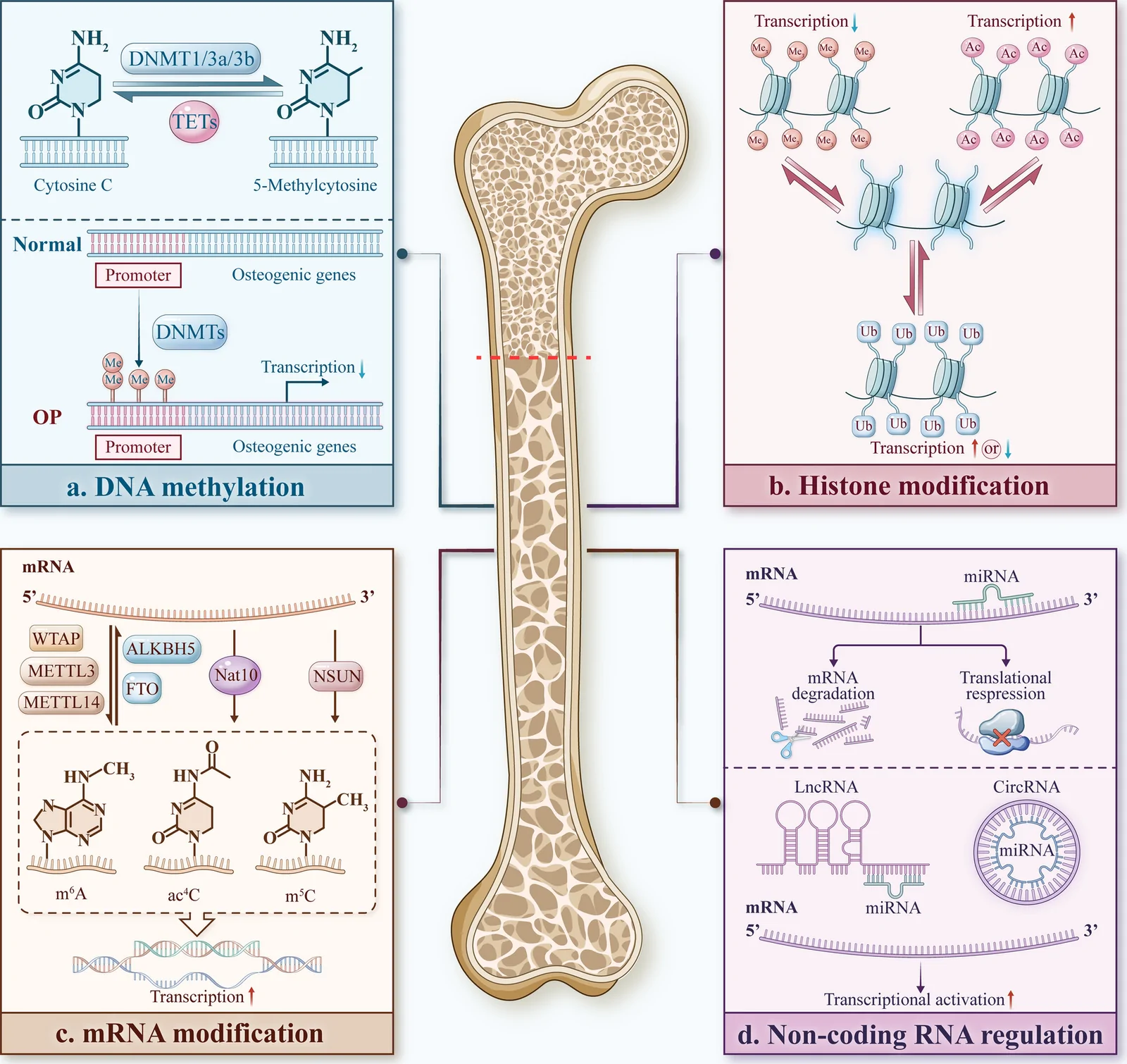
The regulatory processes of epigenetic modifications. **a** The balance of DNA methylation is dynamically maintained by DNA methyltransferases (DNMT1/3a/3b) and TET dioxygenases. Under normal physiological conditions, the promoters of osteogenic genes remain unmethylated, allowing for active transcription. However, in osteoporosis, aberrant DNMT activity leads to the hypermethylation of these promoters, resulting in the transcriptional silencing of osteogenic genes. **b** Histone post-translational modifications at the N-terminal tails promote changes in chromatin structure, thereby regulating gene expression. Generally, specific histone methylations are associated with transcriptional repression, whereas histone acetylation relaxes chromatin to enhance transcription. Meanwhile, histone ubiquitination exerts a dual role, mediating either transcriptional activation or silencing depending on the context. **c** Dynamic mRNA modifications, such as m6A, ac4C, and m5C, regulate gene expression at the post-transcriptional level. These modifications are controlled by specific writers (such as METTL3/14, Nat10, and NSUN) and erasers (including ALKBH5 and FTO), which ultimately determine mRNA fate by influencing its stability, translation, and splicing. **d** Non-coding RNAs, particularly miRNAs, lncRNAs, and circRNAs, orchestrate complex post-transcriptional regulatory networks. Specifically, miRNAs directly bind to target mRNAs to induce transcript degradation or translational repression. LncRNAs and circRNAs predominantly function as competitive miRNA sponges. By sequestering miRNAs, they relieve miRNA-mediated repression and consequently upregulate downstream target gene expression

DNA methylation refers to the transfer of a methyl group from S-adenosylmethionine to the fifth carbon atom of cytosine under the catalysis of DNA methyltransferase (DNMT) to form 5-methylcytosine (5-mC) (Fig. 1a) [25]. In mammalian cells, this modification predominantly occurs at CpG dinucleotides, known as CpG methylation [26]. As a highly conserved epigenetic mark, DNA methylation plays diverse and context-dependent roles in gene regulation, chromatin organization, and the maintenance of genome stability [27].

Mechanistically, hypermethylation of CpG island promoters is typically associated with long-term transcriptional silencing, whereas promoter-associated CGIs usually remain hypomethylated to permit gene expression. Conversely, gene body methylation is generally associated with the prevention of spurious intragenic initiation and active transcription. Furthermore, differential methylation at enhancers and boundary elements, such as CCCTC-binding factor (CTCF) binding sites, dynamically modulates tissue-specific gene activation and three-dimensional chromatin architecture [28–30].

DNA methyltransferases are mainly divided into three types, DNMT1, DNMT2 and DNMT3 [31]. In mammals, DNMT1 and DNMT3 are key enzymes involved in DNA methylation [32]. Among them, DNMT1 is mainly associated with the maintenance of DNA methylation, while DNMT3 plays a key role in de novo methylation [33]. DNMT is involved in the development of a variety of diseases, so it has become an important target for the treatment of a variety of diseases, such as leukemia [34, 35], gastric cancer [36], oral cancer [37–39], hepatocellular carcinoma [40], and so on.

DNA methylation is a relatively stable epigenetic modification. However, it can be reversed through two main demethylation pathways. Passive demethylation occurs during DNA replication when the maintenance activity of DNMT1 is reduced or absent, leading to the gradual dilution of methylation marks [41, 42]. In contrast, active demethylation is mediated by the ten–eleven translocation (TET) family of dioxygenases, including TET1, TET2 and TET3 [43], which catalyze the stepwise oxidation of 5-mC to 5-hydroxymethylcytosine, 5-formylcytosine, and 5-carboxylcytosine, followed by base excision repair–mediated replacement [44–46]. For example, during preimplantation embryo and primordial germ cell development, DNA demethylation can actively establish a pluripotent state and erase specific imprints of parental origin [47, 48].

### Histone modification

Histones H1, H2A, H2B, H3, and H4 are positively charged protein complexes that can interact with negatively charged DNA [49]. The interaction between the octamer composed of H2A, H2B, H3 and H4 molecules and a 147 bp DNA fragment leads to chromatin compaction and assembly into nucleosomes, which are the basic chromatin units [50]. Histone post-translational modifications occur at the N-terminal positions of these nuclear proteins, thereby promoting changes in chromatin structure [51]. These modifications include methylation, acetylation, phosphorylation, ubiquitination, sumoylation, glycosylation, and ADP ribosylation (Fig. 1b) [52]. Histone modifications at enhancer and promoter sites can regulate gene expression [53]. These modifications are promoted by specific enzymes with substrate specificity for histones, such as HATs and HDACs [54], histone methyltransferases and histone demethylases [55], histone kinases and histone phosphatases [56], and so on.

Histone methylation usually occurs at lysine and arginine residues in the N terminus of histones [57]. Among them, lysine methylation sites in mammalian cells include K4, K9, K27, K36 and K79 on H3, and K20 on H4 [58]. In general, trimethylation of H3K4, H3K36, and H3K79 is mainly associated with transcriptional activation, while trimethylation of H3K9, H3K27, and H4K20 is usually associated with transcriptional repression or silencing [59]. Recent advances have extensively decoded the functional complexity of histone arginine methylation in gene expression. Specifically, symmetric modifications such as H3R8me2s and H4R3me2s are classically associated with transcriptional repression [60], whereas asymmetric methylation deposited by enzymes like CARM1/PRMT4 promotes transcriptional activation [61].

Histone acetylation neutralizes the positive charges of lysine residues within the N-terminal tail protruding from the nucleosome. This weakens their attraction to negatively charged DNA, leading to the relaxation of chromatin structure [57] and thereby facilitating the binding of transcription factors to enhance gene transcription [62]. To date, more than 40 specific lysine residues across the four core histones—H2A, H2B, H3, and H4—have been identified as potential acetylation sites [63, 64].

Histone ubiquitination refers to the covalent attachment of the 76-amino-acid ubiquitin protein to specific lysine residues on histones [65]. This enzymatic cascade requires the sequential orchestration of ubiquitin-activating enzymes (E1), ubiquitin-conjugating enzymes (E2), and ubiquitin ligases (E3), which ultimately links ubiquitin molecules to histones to form polyubiquitin chains [66]. All histones can undergo ubiquitination, with H2A and H2B being the most common targets [57]. Functionally, H2A and H2B ubiquitination play pivotal roles in modulating critical nuclear processes, including transcription initiation and elongation, gene silencing, and DNA repair [67].

Histone post-translational modifications are influenced by external environmental factors and the metabolic state of the organism. Aberrant regulation of these modifications has been implicated in the onset and progression of various diseases, including inflammatory disorders, cancer, cardiovascular and renal diseases, metabolic syndromes, and neuropsychiatric disorders [68].

### mRNA modification

mRNA modifications refer to chemical alterations of mRNA molecules that influence their stability, translation, and splicing, thereby regulating gene expression at the post-transcriptional level [69]. These modifications are dynamically controlled by three classes of proteins, writers, erasers, and readers, which catalyze the addition, removal, and recognition of chemical marks to determine mRNA fate and downstream gene expression [70]. To date, more than 170 types of chemical modifications have been identified in cellular RNAs, among which approximately 20–30 have been reported to occur in mRNA [71]. Among these, N6-methyladenosine (m6A), N4-acetylcytosine (ac4C), and 5-methylcytosine (m5C) modifications are known to participate in the regulation of bone metabolism (Fig. 1c) [72–74].

m6A is the most prevalent internal modification in eukaryotic mRNA and is highly conserved across species ranging from yeast to mammals [75]. m6A is a chemical modification generated by the addition of a methyl group to the nitrogen-6 position of adenosine [76]. The methyltransferase complex, comprising METTL3, METTL14, and WTAP, functions as the core writer machinery that catalyzes m6A installation on target transcripts [77]. The demethylases FTO and ALKBH5 serve as erasers, mediating the reversible removal of m6A to ensure dynamic regulation of RNA methylation [78]. m6A readers, including YTH domain family proteins (YTHDF1–3 and YTHDC1–2), insulin-like growth factor 2 mRNA-binding proteins (IGF2BP1–3), and hnRNPs, interpret m6A marks to modulate mRNA splicing, export, translation efficiency, and decay [79]. Through the coordinated actions of these regulators, m6A exerts precise spatiotemporal control over gene expression, thereby influencing diverse cellular processes.

ac4C is an evolutionarily conserved RNA modification generated by the acetylation of the amino group at the N4 position of cytidine. N-acetyltransferase 10 (NAT10) is currently the only known writer enzyme for mRNA ac4C [80]. Emerging evidence reveals that ac4C exerts region-specific effects on mRNA fate, dictating transcript stability and translational output depending on its precise spatial localization [81]. Within the CDS, ac4C enhances mRNA stability by forming stabilizing hydrogen bonds with internal N4-acetyl groups [82]. Specifically, ac4C at the wobble position facilitates the recognition of cognate tRNAs. This modification accelerates translation elongation and simultaneously protects the mRNA from co-translational decay pathways that are typically triggered at non-optimal codon contexts [81]. In contrast to its promotional role in the CDS, ac4C deposition within the 5′ UTR represses protein synthesis [82, 83]. Acetylation events located near strong AUG initiation codons, such as those within the Kozak sequence, competitively inhibit the use of annotated start codons and facilitate upstream initiation, thereby suppressing canonical translation initiation [81–84]. Furthermore, recent transcriptomic mapping has identified ac4C modifications within the 3′ UTR, where they also play essential roles in reinforcing mRNA stability and modulating translation [81]. Mechanistically, this terminal acetylation shields the mRNA from degradation, potentially by altering interactions with 3′ UTR regulatory elements or exonucleases, thereby ensuring robust downstream protein expression [85]. Despite these robust functional insights, the broader epitranscriptomic regulatory network of ac4C remains partially obscured. While SIRT7 has been identified as a putative deacetylase (eraser) for ribosomal RNA, specific erasers and reader proteins dedicated to ac4C on mRNA have yet to be definitively identified [85]. Consequently, discovering supplementary writers, alongside definitive readers and erasers, is currently a major focus for advancing our understanding of ac4C [86].

m5C is another evolutionarily conserved RNA modification characterized by the addition of a methyl group to the fifth carbon of the cytosine ring [87]. This reaction is catalyzed by RNA cytosine methyltransferases, primarily including members of the NSUN family and DNMT2, which together act as the principal writers responsible for m5C deposition on various RNA species [88]. Although the enzymatic demethylation of m5C in RNA is not yet fully elucidated, the dioxygenase TET family has been proposed to function as potential erasers by catalyzing the oxidation of m5C to hm5C [89]. In addition, the ALYREF and YBX1 have been identified as readers that selectively recognize m5C-modified transcripts and mediate their nuclear export or enhance mRNA stability, respectively [90]. Functionally, m5C modification regulates mRNA export, stability, and translation, thereby participating in diverse cellular processes such as stem cell differentiation, stress response [87], and tumorigenesis [91].

### Non-coding RNA

Non-coding RNAs are RNA molecules without protein coding ability [92]. Non-coding RNAs include long non-coding RNA (lncRNA), microRNA (miRNA), pseudogenes, circular RNA (circRNA), small interfering RNA, small nucleolar RNA, ribosomal RNA and transfer RNA [93]. Among them, lncRNA, miRNA and circRNA are involved in post transcriptional regulation [94]. Non-coding RNAs can regulate gene expression by inhibiting the translation of their mRNA targets (Fig. 1d) [95]. The categories of non-coding RNAs involved in the regulation of bone metabolism include miRNAs, lncRNAs, and circRNAs [96].

LncRNAs are transcripts longer than 200 nucleotides. Their mechanism of action involves interacting with chromatin-remodeling machinery to modulate downstream transcription, acting as miRNA sponges to relieve miRNA-mediated repression, and functioning as decoys for transcription factors to suppress target gene expression [97].

MicroRNAs are endogenous single-stranded RNAs approximately 19–23 nucleotides in length. A small subset of miRNAs can bind with perfect complementarity to their target mRNAs, thereby inducing direct mRNA cleavage and degradation [98]. However, the vast majority of miRNAs exhibit only partial complementarity to the 3' UTR of their target mRNAs, which results in the repression of translation rather than direct cleavage, preventing the synthesis of the target proteins [99].

CircRNAs are circular transcripts with hundreds or thousands of nucleotides in length, which are usually formed by alternative splicing of pre-mRNA [100]. CircRNAs can function as transcriptional regulators, miRNA sponges, or protein baits, thereby exerting diverse effects on gene expression [101]. Specifically, they can interact with transcriptional machinery to either activate or repress transcription, act as miRNA sponges to relieve miRNA-mediated repression and enhance target gene expression, or sequester regulatory proteins to modulate downstream signaling and transcriptional outcomes [102]. CircRNAs can be divided into four categories: exonic circRNAs, intronic circular circRNAs, exon intron circRNAs and intergenic circRNAs [103]. Circular RNAs have higher tolerance to exonucleases due to their special circular covalent bonding structure [104]. Due to its conservation, abundance and tissue specificity, circRNAs can play the role of special molecular markers in some diseases [105].

## Mechanisms of epigenetic modifications in different types of osteoporosis

Osteoporosis comprises multiple etiological subtypes. Accumulating evidence indicates that epigenetic modifications serve as key molecular links between these disease-specific cues and dysregulated gene expression in bone cells, as shown in Table 1. This section summarizes how these epigenetic mechanisms drive the development of different types of osteoporosis.

**Table 1:** Mechanisms of epigenetic modifications in Osteoporosis

Osteoporosis	Epigenetics	Mechanism	Function	Study model	Refs
Senile osteoporosis	DNA methylation	DNMT1–-Rora, Fgfr2, Ihh	Osteogenesis ↓	Human & Mouse	[106]
		DNMT3b–-Gal-1	Osteogenesis ↓	Mouse	[107]
		Bmi-1–-DNMT3a–-Runx2	Osteogenesis ↓	Mouse	[108]
	Histone modification	α-KG–-H3K9me3, H3K27me3–-osteogenic related genes	Osteogenesis ↓	Mouse	[109]
		NAP1L2–-SIRT1–-H3K14Ac–-osteogenic related genes	Osteogenesis ↓	Human & Mouse	[110]
		HDAC6–-H3K9/K14Ac, H4K12Ac–-Runx2	Osteogenesis ↓	Mouse	[111]
		CIC–-nuclear acetyl CoA–-H3K27Ac–-osteogenic related genes	Osteogenesis ↓	Mouse	[112]
	mRNA modification	METTL3, IGF2BP2–-m6A–-Slc1a5 mRNA	Osteogenesis ↓	Human & Mouse	[113]
		ALKBH5–-m6A–-VDAC3 mRNA	Osteogenesis ↓	Human	[114]
		METTL3–-m6A–-SIRT1 mRNA	Osteogenesis ↓	Mouse	[115]
	Non-coding RNA	miR-31–-Frizzled-3 mRNA–-Wnt	Osteogenesis ↓	Human	[116]
		miR-214-3p–-ATF4 mRNA	Osteogenesis ↓	Human & Mouse	[117]
		ATF2–-lncRNA NEAT1–-SIRT3/SOD2, BNIP3L, BMP2K, PPARG, CSF1	Osteogenesis ↓ Osteoclastogenesis ↑	Human	[118]
		lncRNA HOTAIR–-H3K27me3–-Runx2, Sp7	Osteogenesis ↓	Mouse	[119]
Postmenopausal osteoporosis	DNA methylation	DNMT3b–-KLF5–-β-catenin	Osteogenesis ↓	Mouse	[120]
		TET1, TET2–-P2rX7–-miR-297 a-5p, miR-297 b-5p, miR-297c-5p–-Runx2	Osteogenesis ↓	Mouse	[121]
		DNMT1, DNMT3a, DNMT3b–-Gpx4	Osteoblast ferroptosis ↑	Mouse	[122]
		TET2–-BCL2–-BECN1	Osteoclastogenesis ↑	Mouse	[123]
	Histone modification	MMP-9–-CS–-mitochondrial citrate–-acetyl CoA	Osteogenesis ↓	Mouse	[124]
		EZH2–-H3K27me3–-genes involved in antioxidant defense and iron homeostasis	Osteoblast ferroptosis ↑	Mouse	[125]
		KDM5a–-H3K4me3–-miR-495–-SKP2–-Runx2	Osteogenesis ↓	Mouse	[126]
	mRNA modification	METTL14–-m6A–-SMAD1 mRNA	Osteogenesis ↓	Human & Mouse	[127]
		NAT10–-ac4C–-NFATc1 mRNA	Osteoclastogenesis ↑	Human & Mouse	[128]
		NAT10–-ac4C–-Runx2 mRNA	Osteogenesis ↓	Mouse	[129]
		CX3CL–-m5C–-NSUN5 mRNA	Osteoclastogenesis ↑	Mouse	[130]
	Non-coding RNA	lncRNA meg3–-microRNA miR-133 a-3p	Osteogenesis ↓	Human & Mouse	[131]
		miR-542-3p–-SFRP1–-Wnt	Osteogenesis ↓	Human & Mouse	[132]
		GATA4–-lncRNA malat1–-KHSRP–-Nedd4–-Runx1	Osteogenesis ↓	Human & Mouse	[133]
		CircRNA hsa_circ_0006859–-miR-642-5p, miR-483-3p–-EFNA2, DOCK3–-Wnt/β-catenin	Osteogenesis ↓	Human & Mouse	[134]
		lncRNA snhg1–-PTBP1–-DNMT1–-OPG	Osteogenesis ↓	Human & Mouse	[135]
		lncRNA dancr, miR-320 a–-Wnt/β-catenin	Osteogenesis ↓	Human & Mouse	[136]
		lncRNA Xist–-miR-450 b–-FBXW7	Osteogenesis ↓	Human & Mouse	[137]
		miR-344d-3p–-DNMT3a	Osteogenesis ↓	Mouse	[138]
Glucocorticoid Induced Osteoporosis	DNA methylation	Wnt/β-catenin–-DNMT3a, DNMT3b–-CEBPA	Osteogenesis ↓	Mouse	[139]
		ACE–-DNMT–-RAS	Osteogenesis ↓	Rat	[140]
	Histone modification	GRα, miR-130 a-5p–-HDAC4–-H3K27Ac–-IGF1	Osteogenesis ↓	Rat	[141]
		HDAC11–-H3K9Ac–−11β-HSD2	Osteogenesis ↓	Rat	[142]
	mRNA modification	METTL3–-m6A–-FNDC5 mRNA–-FNDC5/Irisin	Osteogenesis ↓	Mouse	[143]
	Non-coding RNA	circRNA HIPK3–-miR-124	Osteogenesis ↓	Human	[144]
		lncRNA OIP5-AS1–- miR-335-5p	Osteogenesis ↓	Mouse	[145]
		lncRNA xr877193.1–-PI3K/AKT	Osteoblast ferroptosis ↑	Mouse	[146]
		miR-101-3p–-PTGS2	Osteoclastogenesis ↑	Rat	[147]
Diabetic osteoporosis	DNA methylation	DNMT1–-CALCA	Osteogenesis ↓	Human	[148]
		DNMT1, DNMT3a–-Wnt	Osteogenesis ↓	Rat	[149]
		DNMT3a–-Wnt/β-catenin–-Runx2, OPN	Osteogenesis ↓	Mouse	[150]
		DNMT1, DNMT3a–-Gpx4	Osteoblast ferroptosis ↑	Mouse	[15]
	Histone modification	KDM6b–-H3K27me3–-Wnt/β-catenin	Osteogenesis ↓	Human & Rat	[151]
		H3K27me3–-osteogenic genes H3K27ac–-osteogenesis-inhibitory genes	Osteogenesis ↓	Mouse	[152]
		Ezh2–-H3K27me3	Osteogenesis ↓	Rat	[153]
	mRNA modification	ALKBH5–-m6A–-FGF21 mRNA	Osteogenesis ↓	Human	[154]
		METTL3–-m6A–-ASK1 mRNA–-p38	Osteoblast ferroptosis ↑	Rat	[155]
		ELF1–-IGF2BP2–-m6A–-IFITM3 mRNA	Osteoblast ferroptosis ↑	Mouse	[156]
		TLR4–-FTO–-m6A–-Osteoclastogenic and inflammatory genes	Osteoclastogenesis ↑	Rat	[157]
	Non-coding RNA	lncRNA AK137033–-SFRP2–-Wnt/β-catenin	Osteogenesis ↓	Mouse	[158]
		G9a–-LINC00657–-miR-204-5p–-IGFBP5	Osteogenesis ↓	Rat	[159]
		miR-702-5p–-OGN–-Runx2	Osteogenesis ↓	Rat	[160]
Disuse osteoporosis	DNA methylation	DNMT3b–-sonic hedgehog	Osteogenesis ↓	Mouse	[161]
	Histone modification	H3K9me3, H3K27me3–-ALP, OPN, Runx2	Osteogenesis ↓	Rat	[162]
	Non-coding RNA	circ-ITCH–-IGF2BP2–-BRCA1–-HOXC10	Osteogenesis ↓	Human & Rat	[163]
	mRNA modification	IGF2BP1–-m6A–-Runx2 RNA	Osteogenesis ↓	Mouse	[164]
Cancer-associated bone loss	mRNA modification	NSUN2–-m5C–-KDM6B mRNA–-KDM6B–-NUMB–-Notch/NID1–-RANKL	Osteoclastogenesis ↑	Human & Mouse	[165]
		YTHDF1–-m6A–-EZH2, CDH11 mRNA–-LYN–-SLUG, MEK/ERK	Osteogenesis ↓ Osteoclastogenesis ↑	Human & Mouse	[166]
	Non-coding RNA	miR-5112, miR-1963–-Parp1–-Hoxa1	Osteogenesis ↓	Human & Mouse	[167]
		NSUN2, YBX1–-m5C–-lncRNA MALAT1 mRNA–-miR-17–-RANKL–-AKT, MAPK	Osteoclastogenesis ↑	Human & Mouse	[168]
		lncRNA H19–-miR-532-3p–-E2F7–-EZH2–-H3K27me3–-PTEN–-Akt/mTOR	Osteogenesis ↓ Osteoclastogenesis ↑	Human	[169]
Inflammatory osteoporosis	mRNA modification	FTO–-m6A	Osteogenesis ↓	Mouse	[170]
		Nat10–-ac4C–-Fos mRNA–-MAPK	Osteoclastogenesis ↑	Mouse	[171]
	Non-coding RNA	circAtp9b–-miR-17–92 a	Osteogenesis ↓	Human	[172]
		lncRNA MIAT–-miR let-7i-5p–-CKIP-1	Osteogenesis ↓	Rat	[173]

↑ indicates upregulation or promotion

↓ indicates downregulation or inhibition

### Senile osteoporosis

**Fig. 2:**
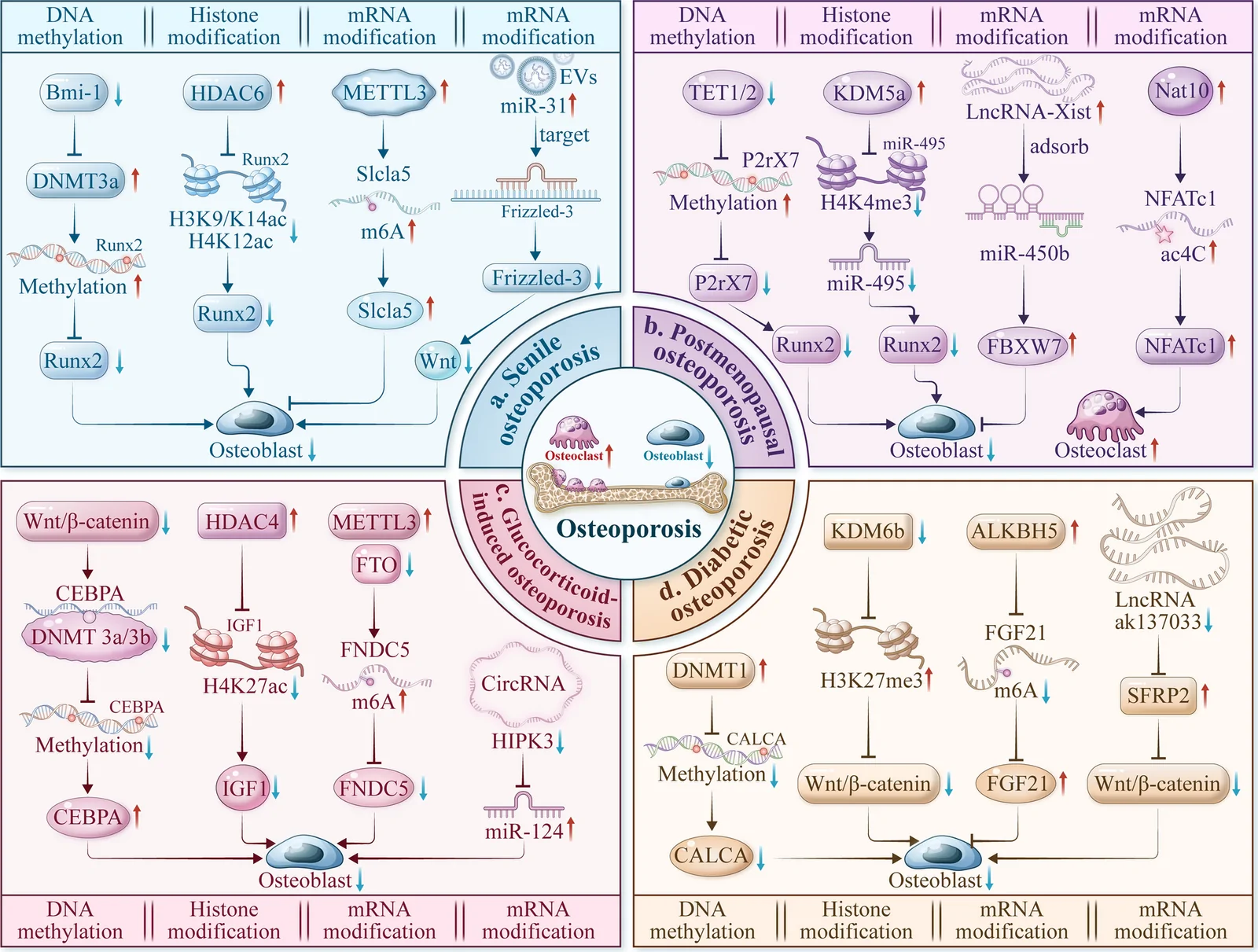
Mechanisms of epigenetic modifications in Osteoporosis. **a** In senile osteoporosis, reduced Bmi-1 and increased HDAC6 suppress Runx2 transcription through DNA hypermethylation and loss of activating histone marks, respectively. Meanwhile, elevated METTL3 enhances m6A modification of Slc1a5, and exosomal miR-31 inhibits Frizzled-3–mediated Wnt signaling. Together, these alterations impair osteoblast formation. **b** In postmenopausal osteoporosis, reduced TET1/2 and increased KDM5a suppress Runx2 transcription by promoting DNA methylation and inhibiting H3K4me3, respectively, while upregulated lncRNA Xist acts through the miR-450b/FBXW7 axis. Collectively, these alterations impair osteoblast differentiation. Meanwhile, elevated NAT10 enhances ac4C modification of NFATc1, promoting osteoclast differentiation. **c** In glucocorticoid-induced osteoporosis, suppressed Wnt/β-catenin signaling enhances DNMT3a/3b-mediated DNA methylation of CEBPA, while elevated HDAC4 reduces H4K27ac at the IGF1 locus. Meanwhile, elevated METTL3 together with reduced FTO increases m6A modification of FNDC5, and circHIPK3 downregulation elevates miR-124 levels. Together, these alterations impair osteoblast differentiation. **d** In diabetes-associated osteoporosis, DNMT1 upregulation suppresses CALCA expression through DNA methylation, while KDM6b downregulation elevates H3K27me3 levels, leading to inhibition of Wnt/β-catenin signaling. Meanwhile, ALKBH5-mediated reduction of m6A enhances FGF21 expression, and loss of lncRNA AK137033 increases SFRP2, further suppressing Wnt/β-catenin signaling. Collectively, these alterations impair osteoblast formation

Senile osteoporosis is an age-driven skeletal deterioration characterized by skeletal stem cell exhaustion, excessive bone marrow adiposity, and the accumulation of senescent cells [174, 175]. While the pathogenesis of senile osteoporosis is multifactorial, the dysregulation of epigenetic regulatory networks acts as a core driver, encompassing alterations in DNA methylation, histone modifications, mRNA modifications, and non-coding RNA expression, as summarized in Fig. 2a.

#### DNA methylation

During aging, the expression of DNMTs becomes dysregulated, thereby significantly impairing osteogenic differentiation potential. A recent study demonstrated that the expression level of DNMT1 protein is increased not only in chondrocyte and bone progenitor cells of aging mouse models in vivo, but also in clinical bone biopsies from the femoral trochanters of patients with senile osteoporosis [106]. Subsequent in vitro mechanistic assays revealed that in MSCs, elevated DNMT1 leads to increased methylation levels in the promoter regions of Rora, Fgfr2, and Ihh genes, which in turn inhibits the expression of these genes and ultimately impairs osteogenic differentiation [106]. Similarly, in preclinical models, DNMT3b expression was upregulated in BMSCs derived from aged mice. This upregulation increased DNA methylation in the promoter region of the galectin-1 (Gal-1) gene, facilitating the recruitment of methyl-CpG binding protein 2 (MeCP2) to this region. Consequently, MeCP2 binding impeded the β-catenin-mediated transcriptional activation of Gal-1, ultimately suppressing the osteogenic differentiation of mouse BMSCs in vitro [107]. Beyond the dysregulated expression of DNMTs themselves, accumulating evidence indicates that age-related disruption of upstream epigenetic regulators also contributes to excessive DNA methylation and impaired osteogenesis. Under physiological conditions, Bmi-1 forms a complex with RING1B and promotes the ubiquitination and proteasomal degradation of DNMT3a, thereby limiting DNA methylation at the Runx2 promoter and sustaining Runx2 expression. In aged mouse BMSCs, reduced Bmi-1 expression disrupts this regulatory axis, leading to DNMT3a accumulation, increased DNA methylation at the Runx2 promoter, and subsequent transcriptional repression of Runx2, which ultimately inhibits osteogenic differentiation and accelerates skeletal aging (Fig. 2a) [108]. Overall, these findings indicate that age-associated dysregulation of DNMTs and their upstream regulators drives aberrant DNA hypermethylation of key osteogenic genes, thereby repressing osteogenic transcriptional programs and contributing to the pathogenesis of senile osteoporosis.

#### Histone modification

Aging-related metabolic alterations lead to dysregulation of histone modifications, thereby impairing the osteogenic differentiation potential of MSCs. In aging mouse models, the age-related decline of α-ketoglutarate (α-KG) compromises the activity of α-KG–dependent histone demethylases, resulting in the accumulation of H3K9me3 and H3K27me3 at osteogenic gene loci and subsequent osteogenic dysfunction of BMSCs [109]. Concurrently, clinical observation revealed that, compared with age-matched healthy controls, the expression of nucleosome assembly protein 1‐like 2 (NAP1L2) was increased in BMSCs from elderly patients with osteoporosis [110]. Subsequent preclinical mechanistic studies demonstrated that NAP1L2 recruits the histone deacetylase SIRT1 to deacetylate H3K14Ac, an active marker in the promoter region of osteogenesis-related genes, thereby inhibiting the osteogenic differentiation of BMSCs [110]. Similarly, in preclinical studies, HDAC6 expression was markedly upregulated in BMSCs from aged mice. HDAC6 was enriched at the Runx2 promoter region, leading to reduced acetylation levels of histone H3K9/K14 and H4K12, thereby repressing Runx2 transcription and impairing the osteogenic differentiation potential of BMSCs (Fig. 2a) [111]. In addition, in aged mouse MSCs, the lysosomal degradation of citrate carrier (CIC) is enhanced, resulting in impaired export of mitochondrial acetyl CoA to the nucleus. The reduction of nuclear acetyl CoA reduces histone H3K27Ac levels in the promoter and enhancer regions of osteogenesis related genes, which in turn reduces chromatin accessibility and ultimately inhibits the osteogenic differentiation ability of MSCs [112]. Taken together, age-related metabolic decline and dysregulated histone-modifying enzymes cooperatively disrupt the epigenetic activation of osteogenic programs, thereby driving impaired MSC osteogenesis and the progression of senile osteoporosis.

#### mRNA modification

During osteoblast senescence, dynamic regulation of m6A modification plays a pivotal role in maintaining cellular homeostasis and osteogenic potential. Clinical observations revealed that the expression of the m6A reader protein IGF2BP2 is upregulated in patients with senile osteoporosis. Subsequent mechanistic studies in mouse models demonstrated that during osteoblast senescence, both IGF2BP2 and the methyltransferase METTL3 are upregulated. METTL3 mediates the m6A modification of Slc1a5 mRNA, which is subsequently recognized and bound by the enriched IGF2BP2. This interaction enhances the stability and expression of Slc1a5 mRNA. Elevated Slc1a5 levels accelerate osteoblast senescence, ultimately impairing osteogenesis and exacerbating age-related bone loss (Fig. 2a) [113]. Similarly, clinical data highlighted a decrease in VDAC3 expression in elderly osteoporotic patients. Further in vitro mechanistic validation using etoposide-induced senescent human BMSCs revealed that ALKBH5 expression is downregulated. This downregulation results in elevated m6A modification, reduced stability, and decreased expression of VDAC3 mRNA. The downregulation of VDAC3 promotes human BMSC senescence and inhibits osteogenic differentiation, thereby contributing to the development and progression of osteoporosis [114]. Conversely, in preclinical mouse models of oxidative stress–induced osteoblast senescence, METTL3 expression is decreased, leading to reduced m6A modification and stability of SIRT1 mRNA, which in turn suppresses SIRT1 expression. The decline in SIRT1 further exacerbates osteoblast senescence and diminishes osteogenic activity [115]. Collectively, these findings highlight that m6A methylation–mediated epitranscriptomic regulation modulates the stability and expression of osteogenesis-related genes, thereby playing an essential role in osteoblast senescence and osteoporosis pathogenesis.

#### Non-coding RNA

Aging-associated disruption of intercellular communication induces dysregulation of non-coding RNAs, thereby contributing critically to the inhibition of bone formation observed in osteoporosis. In human primary cell in vitro models, senescent endothelial cells release microvesicles enriched in miR-31. When internalized by human ADSCs, miR-31 targets and suppresses Frizzled-3, thereby inhibiting Wnt signaling activation and reducing osteogenic differentiation (Fig. 2a) [116]. Similarly, clinical data showed that the level of osteoclast-derived exosomal miR-214-3p increases with age in the serum, serum exosomes, and bone tissue of elderly individuals (including low-energy fracture patients and age-matched controls). Subsequent in vivo mouse models confirmed that this miRNA is transferred to osteoblasts, inhibiting osteoblast activity and bone formation by targeting the 3'-UTR of ATF4 mRNA [117]. Furthermore, clinical observations identified an upregulation of the lncRNA NEAT1 in patients with senile osteoporosis. Further in vitro validation revealed that lncRNA NEAT1 impairs osteogenesis by inducing severe mitochondrial dysfunction and cellular senescence. Meanwhile, it promotes osteoclastogenesis by upregulating CSF1 expression and facilitating its exosome-mediated transfer to osteoclast precursors, thereby aggravating bone metabolic imbalance [118]. Similarly, in mouse models of senile osteoporosis, the upregulated lncRNA HOTAIR suppresses BMSC osteogenesis via EZH2-mediated H3K27me3 silencing of key osteogenic genes, such as Runx2 and Sp7 [119]. As a whole, aging-associated alterations in intercellular communication and stress signaling reprogram the non-coding RNA network in the bone microenvironment, thereby suppressing osteoblast function, enhancing osteoclast activity, and ultimately driving bone formation failure and skeletal imbalance in senile osteoporosis.

### Postmenopausal osteoporosis

PMOP is primarily caused by estrogen deficiency following menopause [176]. Estrogen deficiency triggers extensive epigenetic remodeling, including aberrant DNA methylation, altered histone modification patterns, dysregulated mRNA modifications, and disrupted non-coding RNA expression [177]. These epigenetic alterations affect the transcription and post-transcriptional regulation of key genes involved in osteoblast differentiation, osteoclastogenesis, and bone remodeling, as summarized in Fig. 2b. Exogenous H_2_O_2_ treatment is often used to simulate the oxidative stress state in the bone microenvironment of PMOP.

#### DNA methylation

Estrogen deficiency induces complex epigenetic reprogramming involving abnormal expression of DNMTs and TET family proteins, which in turn profoundly affects bone homeostasis by regulating key genes. Upregulation of DNMT3b expression was observed in ovariectomized (OVX) mice and their H_2_O_2_ treated BMSCs. Elevated DNMT3b promoted the methylation of the promoter region of the zinc finger transcription factor Krüppel-like factor 5 (KLF5), which in turn inhibited KLF5 expression. The downregulation of KLF5 inhibits the nuclear translocation and expression of β-catenin by interacting with the armadillo repeat region of β-catenin, and impairs the osteogenic differentiation ability of MSCs [120]. In addition, compared with normal mice, the expression of TET1 and TET2 in BMSCs of OVX mice was downregulated. The lack of TET1/TET2 inhibited the demethylation of the P2rX7 promoter region, resulting in decreased P2rX7 expression, which in turn inhibited exosome secretion. This causes the accumulation of miR-297 a-5p, miR-297 b-5p, and miR-297c-5p, and finally impairs the osteogenic differentiation of BMSCs by inhibiting the Runx2 signaling pathway (Fig. 2b) [121]. Meanwhile, the osteoblasts in OVX mice exhibit significant ferroptosis characteristics and transcriptional repression of the key ferroptosis regulator Gpx4. This phenomenon is associated with the abnormal elevation of DNMT1, DNMT3a and DNMT3b expression in osteoblasts. These DNMTs lead to hypermethylation of Gpx4 promoter region and inhibit its transcription, thus inducing osteoblast ferroptosis, reducing bone formation, and ultimately contributing to the osteoporotic phenotype [122]. Finally, TET2 expression was elevated in osteoclasts of OVX mice. Elevated TET2 inhibits BCL2 expression, reduces the binding of BCL2 to BECN1, an autophagy protein, and enhances autophagy activity by relieving the inhibition of BECN1 by BCL2, ultimately promoting osteoclast differentiation and bone resorption [123]. Collectively, current preclinical evidence indicates that estrogen deficiency–induced dysregulation of DNMT and TET activities reshapes the epigenetic landscape of bone cells, driving impaired osteogenic signaling, ferroptotic osteoblast loss, and excessive osteoclast activation in mouse OVX models.

#### Histone modification

Estrogen deficiency profoundly reshapes the histone modification landscape in BMSCs and osteoblast-lineage cells, thereby disrupting osteogenic gene expression programs and impairing bone formation. For example, in OVX mice, estrogen deficiency-induced oxidative stress restricts the supply of nuclear acetyl-CoA, reducing global histone acetylation and inhibiting BMSC osteogenesis [124]. Beyond metabolic constraints on acetylation, aberrant histone methylation further exacerbates osteogenic dysfunction. In OVX mice, excessive EZH2-mediated H3K27me3 deposition represses antioxidant and iron homeostasis genes, triggering osteoblast ferroptosis and impairing osteogenesis. Notably, in these in vivo models, inhibiting this epigenetic axis with low-dose valproic acid effectively suppresses ferroptosis and ameliorates bone loss [125]. Additionally, the upregulated histone demethylase KDM5a in OVX models inhibits the transcription of miR-495 by reducing the level of H3K4me3, triggering a downstream cascade that ultimately accelerates the proteasomal degradation of the core osteogenic transcription factor Runx2, thereby inhibiting osteogenesis (Fig. 2b) [126]. In aggregate, findings from animal studies suggest that estrogen deficiency triggers metabolic rewiring and dysregulates chromatin-modifying enzymes to reprogram histone acetylation and methylation, thereby inducing ferroptotic stress, destabilizing Runx2, and ultimately impairing osteogenic differentiation in mouse OVX models.

#### mRNA modification

Dynamic RNA modifications act as crucial epitranscriptomic regulators in estrogen deficiency-induced bone loss. Clinical analyses revealed that METTL14 expression and global m6A levels are downregulated in patients with PMOP. Subsequent mechanistic validation in OVX mice demonstrated that this deficiency leads to reduced m6A methylation of SMAD1 mRNA. This decrease attenuates the recognition and stabilizing effect of the m6A reader protein IGF2BP1 on SMAD1 mRNA, promoting its degradation and reducing its expression, ultimately impairing osteogenic differentiation of mouse BMSCs [127]. Similarly, clinical observations highlighted that in femoral tissues of PMOP patients, NAT10 expression and its catalyzed ac4C modification levels are elevated. Further in vivo validation in OVX mice showed that NAT10 expression is upregulated in bone marrow-derived macrophages (BMDMs), where it mediates ac4C modification of NFATc1 mRNA, thereby enhancing its translational efficiency, promoting osteoclast differentiation and bone resorption, and ultimately accelerating bone loss (Fig. 2b) [128]. Conversely, preclinical mouse data indicated that NAT10 expression is downregulated in BMSCs of OVX mice, leading to reduced ac4C modification of Runx2 mRNA. This reduction diminishes Runx2 mRNA stability and translational efficiency, thereby suppressing osteogenic differentiation and exacerbating bone loss [129]. Moreover, CX3CL1 expression is upregulated in BMDMs of OVX mice, promoting macrophage M1 polarization and osteoclast differentiation, which enhances bone resorption and contributes to bone loss. Overexpression of NSUN5 increases the m5C modification level of CX3CL1 mRNA, thereby reducing its stability and expression, attenuating M1 polarization and osteoclast activation, and significantly ameliorating the OVX-induced osteoporotic phenotype in mice. However, co-overexpression of CX3CL1 and NSUN5 partially offsets the protective effects mediated by NSUN5 [130]. Collectively, these findings indicate that RNA modifications, including m6A, ac4C, and m5C, regulate the stability and translational efficiency of key osteogenic and osteoclastic genes, thereby playing crucial roles in the development and progression of PMOP and its corresponding OVX models.

#### Non-coding RNA

In PMOP, the extensive dysregulation of non-coding RNA network is the core mechanism that impairs the osteogenic differentiation potential of BMSCs. Analysis of clinical samples showed that lncRNA meg3 expression was increased in BMSCs of PMOP patients. In vivo validation in OVX mice demonstrated that Meg3 negatively regulates osteogenic differentiation of mouse BMSCs by promoting microRNA miR-133 a-3p expression [131]. Meanwhile, the expression of multiple inhibitory miRNAs is downregulated in PMOP patients. Coupled with mouse mechanistic studies, it was shown that reduced miR-542-3p expression weakens its inhibitory effect on the 3'-UTR of secreted frizzled related protein 1 (SFRP1) mRNA, resulting in the increased expression of SFRP1, which in turn inhibits the osteogenic differentiation of mouse MSCs [132]. In addition, clinical assessments identified an imbalance of key regulatory factors in the bone tissue of PMOP patients, where the expression of the transcription factor GATA4, lncRNA MALAT1, and the RNA-binding protein KHSRP was downregulated, whereas the E3 ubiquitin ligase NEDD4 was upregulated. Subsequent mouse models elucidated the mechanism, demonstrating that GATA4 activates MALAT1 transcription, and MALAT1 forms a complex with KHSRP to promote Nedd4 mRNA degradation. Elevated Nedd4 expression degrades Runx1, a key osteogenic transcription factor, through ubiquitination, impairing the osteogenic ability of mouse BMSCs [133]. Furthermore, circRNA hsa_circ_0006859 expression is elevated in BMSCs from both PMOP patients and OVX mice, but decreased during in vitro osteogenic differentiation. Mechanistic assays in mice showed that, as a molecular sponge, it adsorbs miR-642 b-5p and miR-483-3p, relieves the inhibition of EFNA2 and DOCK3, and subsequently inhibits the Wnt/β-catenin signaling pathway, ultimately inhibiting the osteogenic differentiation of mouse BMSCs [134]. Another study demonstrated that lncRNA snhg1 expression is elevated in PMOP. In vivo mouse models confirmed that Snhg1 promotes DNMT1 expression by upregulating PTBP1, leading to hypermethylation of the osteoprotegerin (OPG) promoter and its expression inhibition, thereby promoting adipogenic differentiation of mouse BMSCs and inhibiting osteogenesis [135]. lncRNA-dancr and miR-320 a are also found to be jointly upregulated in PMOP patients. Validation in OVX mice revealed that both independently target and inhibit β-catenin expression, suppressing Wnt/β-catenin signaling and osteogenic differentiation [136]. Notably, lncRNA Xist is also highly expressed in PMOP patients. In vivo mechanistic studies in OVX mice showed that it relieves its inhibition of FBXW7 by adsorbing miR-450 b, resulting in FBXW7 upregulation and inhibition of mouse osteogenic differentiation (Fig. 2b) [137]. Finally, shifting to preclinical evidence, the decreased expression of miR-344d-3p in mandibular-derived MSCs from OVX mice relieves its inhibition of the 3'-UTR of DNMT3a mRNA and promotes DNMT3a protein translation, ultimately leading to weakened osteogenic ability and enhanced adipogenic propensity in these mouse MSCs [138]. Taken together, dysregulated lncRNAs, miRNAs, and circRNAs form an integrated pathogenic network in PMOP and its corresponding OVX models. This network silences osteogenic transcription factors and Wnt signaling, ultimately suppressing the osteogenic differentiation of BMSCs.

### Glucocorticoid-induced osteoporosis

GIOP is one of the most common forms of secondary osteoporosis, primarily resulting from prolonged or excessive exposure to glucocorticoids [178]. Beyond their classical genomic and non-genomic effects, glucocorticoids also induce extensive epigenetic reprogramming in bone cells. These epigenetic alterations collectively contribute to the transcriptional repression of osteogenic genes and activation of adipogenic pathways, ultimately leading to reduced bone formation and increased bone resorption in GIOP, as summarized in Fig. 2c.

#### DNA methylation

In preclinical models of GIOP, dexamethasone exposure can reprogram the epigenetic landscape of mouse MSCs, promote their adipogenic differentiation tendency, and inhibit osteogenic differentiation. Compared with the untreated control group, dexamethasone-treated mouse BMSCs and the mouse mesenchymal cell line C3H10T1 showed an increase in the expression level of the adipogenic dominant transcription factor C/EBPα (CEBPA) during osteogenic differentiation. Mechanistic studies revealed that dexamethasone blocked the binding of DNMT3a and DNMT3b to the CEBPA promoter region by inhibiting the Wnt/β-catenin signaling pathway. This process reduces the methylation level of the CEBPA promoter region, thus promoting CEBPA protein expression. The increase in CEBPA levels leads to the shift of BMSCs and C3H10T1 cells toward adipogenic differentiation, while weakening their osteogenic differentiation potential (Fig. 2c) [139]. Furthermore, another in vivo study using a rat model demonstrated that excessive glucocorticoids further inhibit osteogenic differentiation through global epigenetic regulation. Glucocorticoids promote the expression of angiotensin-converting enzyme (ACE) genes by inhibiting the methylation level of the promoter region of ACE-related genes. This process subsequently activates the renin-angiotensin system (RAS) signaling pathway, ultimately inhibiting the osteogenic differentiation ability of rat BMSCs [140]. Consequently, current preclinical evidence indicates that excessive glucocorticoids remodel DNA methylation patterns in mouse MSCs, thereby inhibiting osteogenic differentiation in GIOP models.

#### Histone modification

In preclinical models, prenatal dexamethasone exposure (PDE) impairs offspring's bone formation ability and increases the risk of osteoporosis by reprogramming the epigenetic landscape. PDE can inhibit the osteogenic differentiation potential of BMSCs in offspring rats. Mechanistically, PDE-induced reduction of serum corticosterone (CORT) in offspring suppresses the expression of glucocorticoid receptor α (GRα) and miR-130 a-5p, thereby upregulating HDAC4. This elevated HDAC4 reduces the active histone mark H3K27ac at the insulin-like growth factor 1 (IGF1) promoter, suppressing IGF1 signaling and ultimately impairing the osteogenic differentiation of rat BMSCs. Notably, chronic stress-induced elevation of CORT concentrations can reverse these molecular changes and partially rescue the osteogenic differentiation defects of BMSCs (Fig. 2c) [141]. In addition, further study in rat models demonstrated that PDE also affects fetal bone development through another independent mechanism [142]. In the bone tissue of PDE offspring, H3K9ac levels in the promoter region of the 11-β hydroxysteroid dehydrogenase 2 (11β-HSD2) gene were decreased. Mechanistically, dexamethasone exposure activates the glucocorticoid receptor (GR) by promoting HDAC11 recruitment to the 11β-HSD2 promoter region of fetal osteoblasts. The activated GR-HDAC11 complex catalyzes the deacetylation of H3K9ac at the promoter region of 11β-HSD2, thereby inhibiting its transcription. The decreased expression of 11β-HSD2 weakens its ability to inactivate CORT, leading to the local accumulation of active glucocorticoids (corticosterone) in bone, which ultimately inhibits the osteogenic differentiation of rat BMSCs [142]. Overall, these preclinical findings in rats indicate that dexamethasone exposure disrupts glucocorticoid metabolism and IGF1 signaling through HDAC-dependent histone deacetylation, leading to persistent suppression of osteogenic differentiation in BMSCs and increased susceptibility to osteoporosis in the offspring.

#### mRNA modification

Maternal prednisone exposure during pregnancy induces aberrant epitranscriptomic regulation in the offspring’s skeletal muscle. Specifically, prenatal prednisone treatment upregulates METTL3 and downregulates the demethylase FTO, thereby increasing the m6A modification level of FNDC5 mRNA and reducing its stability and expression. Suppression of the FNDC5/Irisin signaling pathway further impairs mitochondrial autophagy in skeletal muscle, disrupting the muscle–bone axis–mediated regulation of bone formation and ultimately increasing the susceptibility of female mouse offspring to osteoporosis in adulthood (Fig. 2c) [143]. These animal findings suggest that this pathway provides a mechanistic link between glucocorticoid exposure and long-term skeletal vulnerability.

#### Non-coding RNA

Glucocorticoid exposure can induce programmed death and dysfunction of osteoblasts, the mechanism of which involves the dysregulation of key non-coding RNAs. In vitro studies using the human osteoblast cell line OB-6 showed that dexamethasone treatment downregulates circRNA HIPK3, leading to the accumulation of its target miR-124. The elevation of miR-124 enhances the sensitivity of these human osteoblasts to cytotoxicity, and finally induces apoptosis and necrosis (Fig. 2c) [144]. Shifting to mouse models, the expression of lncRNA OIP5-AS1 is upregulated in both GIOP mice and dexamethasone-treated mouse osteoblastic MC3T3-E1 cells. This elevated OIP5-AS1 functions to sponge and suppress its downstream target, miR-335-5p. This dysregulated OIP5-AS1/miR-335-5p axis triggers excessive autophagy and oxidative stress, restraining proliferation and osteogenic differentiation [145]. Furthermore, glucocorticoids drive osteoblast depletion via ferroptosis. Further data indicated that in dexamethasone-treated MC3T3-E1 cells, lncRNA xr877193.1 expression is upregulated. This lncRNA exacerbates cellular oxidative damage and lipid peroxidation by inhibiting the PI3K/AKT pathway, thereby promoting the occurrence of ferroptosis and ultimately compromising osteogenesis [146]. Beyond direct osteoblast impairment, non-coding RNA dysregulation exacerbates GIOP by promoting excessive osteoclastogenesis via intercellular communication. Specifically, in vivo rat models demonstrated that the pathological downregulation of rat BMSC-derived exosomal miR-101-3p removes its suppression on PTGS2, promoting osteoclast hyperactivation and severe bone resorption [147]. Together, glucocorticoid-induced dysregulation of diverse non-coding RNA networks drives multifaceted osteoblast impairment, while simultaneously accelerating osteoclast-mediated bone resorption, thereby profoundly disrupting bone homeostasis in GIOP.

### Diabetic osteoporosis

Diabetic osteoporosis (DOP) is a metabolic bone disorder that occurs as a chronic complication of both type 1 and type 2 diabetes mellitus [179]. Persistent hyperglycemia, insulin deficiency or resistance, and the accumulation of advanced glycation end products (AGEs) impair osteoblast function, enhance osteoclast activity, and deteriorate bone quality, ultimately increasing fracture risk despite normal or elevated bone mineral density. Recent studies indicate that these diabetes-associated alterations are tightly linked to widespread epigenetic dysregulation, encompassing DNA methylation, histone modifications, mRNA modifications, and non-coding RNAs, as summarized in Fig. 2d.

#### DNA methylation

In DOP and associated in vitro models, the abnormal activation of DNMTs induced by AGEs is a key epigenetic mechanism that impairs the osteogenic differentiation of MSCs. Specifically, in vitro studies using human PDLSCs treated with AGEs to simulate the diabetic microenvironment revealed that DNMT1 expression was increased. Elevated DNMT1 catalyzes the hypermethylation of the calcitonin gene-related peptide alpha (CALCA) promoter region, thereby inhibiting CALCA mRNA transcription and ultimately impairing the osteogenic differentiation potential of these human PDLSCs (Fig. 2d) [148]. Similarly, in AGE-treated rat ADSCs, the expression of the receptor for AGEs is upregulated, which in turn promotes the expression of DNMT1 and DNMT3a. The elevation of DNMTs leads to an increase in global DNA methylation levels, inhibiting the activity of the Wnt signaling pathway, and ultimately suppressing the osteogenic differentiation of ADSCs [149]. Consistent with these findings, in ADSCs isolated from DOP mice, the specific elevation of DNMT3a expression increases the DNA methylation load, inhibits the activity of the Wnt/β-catenin signaling pathway, and downregulates the expression of key osteogenic genes Runx2 and osteopontin (OPN), ultimately impairing the osteogenic differentiation ability of these cells [150]. Preclinical in vivo studies further confirmed the central role of DNMTs. In DOP mouse models, abnormally high expression of DNMT1 and DNMT3a led to hypermethylation of the Gpx4 promoter, thereby inhibiting Gpx4 transcription and protein expression. Gpx4 deletion triggered osteoblast ferroptosis, resulting in decreased bone formation and increased bone resorption, which together promoted osteoporosis. Notably, the natural compound salicin VI reverses the hypermethylation status of the Gpx4 promoter and restores Gpx4 expression by binding to DNMT1 and DNMT3a proteins and promoting their lysosomal degradation, ultimately alleviating osteoblast ferroptosis and improving the osteoporotic phenotype in mice [15]. Taken together, diabetes-driven overactivation of DNMT1 and DNMT3a establishes a hypermethylated epigenetic state that silences Wnt/β-catenin signaling, CALCA, and Gpx4. This epigenetic reprogramming thereby promotes osteogenic failure and osteoblast ferroptosis, ultimately driving diabetic osteoporosis.

#### Histone modification

Physiological mechanical stress promotes osteogenic differentiation of PDLSCs through epigenetic reprogramming, a regulatory process impaired by the diabetic microenvironment [151]. Mechanistically, initial in vitro experiments in hPDLSCs revealed that mechanical stress upregulates the expression of the histone demethylase KDM6b. Subsequent in vivo validation in rat models demonstrated that this elevated KDM6b decreases H3K27me3 levels to relieve the transcriptional repression of key target genes, thereby activating the Wnt/β-catenin pathway and promoting osteogenesis. Further in vivo evidence confirmed that exposure to AGEs, which mimics the diabetic pathological state, blunts this mechanosensory response. AGEs reduce the mechanical sensitivity of KDM6b and weaken its stress-induced upregulation. Consequently, the activation of this epigenetic and signaling cascade is aborted, thereby impairing the osteogenic potential of PDLSCs (Fig. 2d) [151]. Beyond AGEs, lipotoxicity induced by a high-fat diet (HFD), another central component of type 2 diabetes and metabolic syndrome, triggers widespread imbalances in histone modifications to further impair osteogenesis. Specifically, in a murine maternal HFD model, this lipotoxic environment drives H3K27me3 enrichment at osteogenic genes and H3K27ac accumulation at osteogenesis-inhibitory genes. Consequently, this dual epigenetic disruption extinguishes osteoblast differentiation and bone formation [152]. The pathogenic impact of this HFD-driven epigenetic silencing is further corroborated in adult rat models, where deterioration of bone quality is linked to the upregulation of the histone methyltransferase Ezh2 and the accumulation of H3K27me3. Notably, targeted intervention with soy isoflavones antagonizes this pathological reprogramming by suppressing Ezh2 overactivity, thereby preventing HFD-induced bone loss [153]. Collectively, the complex diabetic microenvironment, driven by AGEs accumulation and lipotoxicity, disrupts the balance of histone modifications. This epigenetic dysregulation ultimately silences key osteogenic networks and impairs bone formation in DOP.

#### mRNA modification

Dynamic mRNA modifications constitute another pivotal layer of epigenetic regulation governing bone cell fates under diabetic conditions. Based on human in vitro evidence, high glucose upregulates the demethylase ALKBH5 in human BMSCs, reducing m6A methylation on FGF21 mRNA. This hypomethylation limits YTHDF2-mediated degradation, thereby stabilizing FGF21 and ultimately impairing osteogenic differentiation [154]. Similarly, in vivo rat models also exhibit impaired bone formation. In a HGHF–induced DOP rat model, METTL3 expression is significantly upregulated. METTL3 promotes m6A methylation of ASK1 mRNA, which increases its stability and activates the ASK1–p38 signaling pathway. This activation triggers ferroptosis in osteoblasts, inhibits osteogenic differentiation, and ultimately exacerbates bone loss (Fig. 2d) [155]. Furthermore, in vivo STZ/HFD-induced DOP mouse models provide additional evidence for m6A-mediated osteoblast ferroptosis. Under diabetic metabolic stress, ELF1 is activated, which subsequently upregulates the expression of IGF2BP2. This upregulation promotes the binding of IGF2BP2 to m6A modifications, thereby enhancing the mRNA stability of IFITM3. Ultimately, this ELF1-IGF2BP2-m6A-IFITM3 signaling cascade amplifies lipid peroxidation and mitochondrial dysfunction, driving ferroptosis-mediated osteoblast senescence and exacerbating diabetic bone loss [156]. Beyond its detrimental effects on osteogenesis, diabetes-induced m6A dysregulation also drives excessive bone resorption. Specifically, in DOP rat models, aberrant activation of TLR4 promotes FTO-mediated m6A demethylation, thereby upregulating the expression of osteoclastogenic and inflammatory genes and accelerating bone loss. Reversing this pathological process, genetic ablation of TLR4 downregulates the m6A demethylase FTO, restores m6A methylation levels on osteoclast-related transcripts, suppresses osteoclast differentiation, and ultimately attenuates diabetic osteoporosis [157]. Collectively, these findings indicate that dysregulation of m6A methylation plays a crucial role in the pathogenesis of DOP under diverse pathological conditions, disrupting both bone formation and resorption.

#### Non-coding RNA

Non-coding RNAs serve as post-transcriptional networks that dynamically modulate the diabetic bone microenvironment. In ADSCs derived from a mouse model of DOP, the expression level of lncRNA AK137033 is downregulated. This downregulation diminishes DNA methylation at the promoter region of SFRP2, thereby upregulating this antagonist and silencing the osteo-protective Wnt/β-catenin signaling cascade, ultimately suppressing ADSC osteogenic differentiation (Fig. 2d) [158]. Furthermore, in rat models, the aberrant upregulation of the histone methyltransferase G9a in BMSCs impairs bone formation in the diabetic milieu. At the epigenetic level, this excessive G9a activity silences LINC00657, thereby disrupting its ability to sponge miR-204-5p and reducing the expression of IGFBP5, ultimately suppressing the osteogenic potential of these stem cells. Pharmacological inhibition of G9a dismantles this pathological cascade and restores this potential [159]. In parallel, hyperglycemic conditions can also exploit microRNA networks to cripple rat osteoblast functionality. Upon exposure to this high-glucose microenvironment, these osteoblasts aberrantly upregulate miR-702-5p, which in turn suppresses OGN and subsequently downregulates Runx2. Dysregulation of the miR-702-5p/OGN/Runx2 axis blunts both proliferation and mineralization, driving DOP progression [160]. In summary, the dysregulation of these non-coding RNAs drives aberrant bone metabolism within the diabetic microenvironment.

### Other types of osteoporosis

#### Disuse osteoporosis

Disuse osteoporosis is a rapid and severe form of skeletal deterioration induced by the loss of mechanical loading, commonly observed during prolonged bed rest, spinal cord injury, or spaceflight [180]. Mechanistically, mechanical unloading impairs bone formation through epigenetic reprogramming, which serves as a core pathological driver.

In a mouse model of disuse osteoporosis induced by long-term immobilization, the sustained weakening of bone mechanical stress significantly upregulates DNMT3b expression. Elevated DNMT3b catalyzes the hypermethylation of the Sonic hedgehog gene promoter, inhibiting its transcription and repressing hedgehog signaling activity to suppress the osteogenic potential of BMSCs [161]. In vitro studies on rat osteoblasts under simulated microgravity (SMG) demonstrate that mechanical unloading also disrupts bone formation through histone modification dysregulation. SMG treatment increases the deposition of repressive histone marks H3K9me3 and H3K27me3 at the promoters of key osteogenic genes, including ALP, OPN, and Runx2, thereby silencing their transcription and blunting osteogenic differentiation [162].

Furthermore, evidence from both an in vivo rat microgravity model and human BMSCs demonstrates that circ-ITCH is downregulated under disuse conditions, weakening its interaction with the m6A reader IGF2BP2. This attenuation compromises the IGF2BP2-mediated stabilization of BRCA1 mRNA. The resulting reduction in BRCA1 expression impairs the ubiquitination and degradation of the osteogenic suppressor HOXC10, leading to its accumulation and the subsequent inhibition of BMSC osteogenic differentiation [163]. Similarly, in a mouse model of mechanical unloading, global m6A modification levels and IGF2BP1 expression are significantly decreased in osteoblasts, reducing Runx2 mRNA stability and impairing bone formation. Notably, melatonin treatment restores this osteogenic capacity by upregulating IGF2BP1, which enhances its binding to m6A-modified Runx2 mRNA to increase its stability and translation, thereby recovering osteoblast function and alleviating bone loss [164].

Consequently, mechanical unloading remodels DNA methylation, histone marks, and m6A-mediated RNA stability to suppress osteogenic gene expression, thereby promoting disuse-induced bone loss.

#### Cancer-associated bone loss

Cancer-associated bone loss is a devastating skeletal complication primarily triggered by the bone metastasis of solid tumors or hematological malignancies [181]. In this pathological context, epigenetic dysregulation acts as a primary driver, rewriting the transcriptional programs of resident bone cells to facilitate tumor-driven bone destruction.

Clinical evidence reveals aberrant NSUN2 upregulation in human advanced breast cancer (BCa) tissues. In vitro studies further demonstrate that in human BCa cell lines, elevated NSUN2 promotes m5C modifications on KDM6B mRNA and accelerates its degradation via the reader protein PPIA. This depletion impairs NUMB demethylation, unleashing the Notch/NICD1 signaling cascade to upregulate RANKL and promote osteoclast differentiation [165]. Consistent with these clinical findings, the m6A reader protein YTHDF1 is elevated in human BCa bone metastases. In vitro analyses reveal that upregulated YTHDF1 in BCa cell lines enhances the translation of EZH2 and CDH11. Subsequently, EZH2 promotes the transcription of LYN, which activates the SLUG and MEK/ERK pathways. This signaling axis not only facilitates tumor cell migration and osteoblast adhesion but also accelerates osteoclast differentiation, exacerbating osteolytic destruction [166]. Furthermore, in vitro co-culture models show that human prostate cancer cell lines disrupt normal osteoclasts, prompting them to secrete EVs loaded with miR-5112 and miR-1963. These exosomal microRNAs target Parp1 in osteoclasts to exacerbate IL-1β-driven inflammatory osteolysis, and Hoxa1 in osteoblasts to suppress osteoblast differentiation, thereby orchestrating a vicious cycle of tumor-associated bone destruction [167].

Beyond solid tumors, hematological malignancies, such as multiple myeloma (MM), also disrupt bone homeostasis by modulating intricate epigenetic networks. In human MM cell lines, the methyltransferase NSUN2 and the reader protein YBX1 are upregulated. NSUN2 elevates the m5C modification of the lncRNA MALAT1, which is subsequently stabilized by YBX1 and secreted via exosomes. Upon internalization by osteoclast precursors, exosomal MALAT1 sponges miR-17 to upregulate RANKL and activate downstream AKT and MAPK pathways, accelerating osteoclastogenesis [168]. Clinical data indicate that lncRNA H19 is significantly upregulated in MM patients. Functional assays demonstrate that in human MM cell lines, elevated H19 acts as a molecular sponge for miR-532-3p, upregulating E2F7. This subsequently drives the expression of EZH2, which enriches H3K27me3 at the PTEN promoter to silence its expression. This PTEN downregulation hyperactivates the Akt/mTOR signaling pathway, simultaneously inhibiting osteoblastogenesis and driving excessive osteoclast formation [169].

Taken together, these findings indicate that diverse malignancies modulate epigenetic networks to disrupt the balance between osteogenesis and osteoclastogenesis. This disruption establishes a bone microenvironment favorable for tumor bone metastasis. Consequently, deciphering and targeting these intricate epigenetic networks may provide promising therapeutic avenues for halting tumor-associated bone destruction.

#### Inflammatory osteoporosis

Inflammatory osteoporosis is a skeletal complication of systemic autoimmune and autoinflammatory diseases, such as rheumatoid arthritis and inflammatory bowel disease [182]. Under these conditions, the excessive secretion of pro-inflammatory cytokines into the bone microenvironment stimulates osteoclast differentiation and impairs osteoblast function. Crucially, the dysregulation of epigenetic networks serves as a pivotal mechanism mediating this cytokine-driven disruption of bone homeostasis.

In mouse models of inflammatory bowel disease, the SUMOylation of the m6A demethylase FTO is aberrantly elevated in BMSCs, disrupting its normal demethylase activity and leading to an abnormal accumulation of m6A levels on target mRNAs, which ultimately inhibits osteogenic differentiation and exacerbates skeletal deterioration [170]. Beyond m6A modifications, mRNA ac4C acetylation also serves as a potent catalyst for inflammatory osteoclastogenesis. In vitro experiments have demonstrated that upon inflammatory stimulation, the acetyltransferase NAT10 is upregulated and catalyzes the ac4C modification of Fos mRNA, thereby promoting Fos expression. The increase in Fos activates the MAPK pathway, leading to excessive osteoclast formation and aggravating bone resorption [171].

Furthermore, inflammatory stimuli perturb the non-coding RNA networks governing osteoblast survival and function. For instance, clinical data showed that inflammation promotes an increase in circAtp9b expression. Further in vitro validation demonstrated that LPS stimulation of human osteoblasts promotes circAtp9b expression, thereby inhibiting the formation of mature miR-17–92 a, which ultimately promotes osteoblast apoptosis and reduces bone formation [172]. Similarly, lncRNA MIAT is upregulated in rat models of rheumatoid arthritis. Subsequent in vitro experiments verified that lncRNA MIAT acts as a molecular sponge for miR let-7i-5p, alleviating the suppression of its target gene CKIP-1, thereby upregulating CKIP-1 expression to suppress osteoblast differentiation [173].

In summary, the aberrant crosstalk between chronic inflammation and epigenetic networks promotes bone loss by simultaneously suppressing osteogenesis and hyperactivating osteoclastogenesis.

### Limitations and future perspectives of mechanistic research

#### Current mechanistic limitations

Epigenetic modifications in osteoporosis include both well-established mechanisms and emerging hypotheses. DNA methylation, classical histone modifications (encompassing both deacetylation and dynamic methylation), and extensive non-coding RNA networks are well-established hallmarks of osteoporotic bone loss. In contrast, other epigenetic modifications, such as mRNA modifications (m6A, ac4C, m5C) and histone ubiquitination, remain emerging hypotheses, and their precise roles in osteoporosis remain unclear. For instance, there is conflicting evidence regarding the effects of m6A modifications, whose specific roles in osteoporosis depend heavily on the specific cellular context and the repertoire of reader proteins present.

Beyond these mechanistic uncertainties, while many studies have clarified the epigenetic mechanisms underlying various osteoporosis subtypes, significant knowledge gaps remain regarding their spatial and temporal dynamics. Most current studies focus solely on the static epigenetic state at late disease stages, while the early epigenetic initiation state of osteoporosis remains largely unexplored. Furthermore, traditional bulk sequencing approaches obscure the epigenetic heterogeneity among different osteoblast-lineage subpopulations.

#### Future directions for mechanistic research

To overcome these methodological constraints, future mechanistic investigations must prioritize decoding the dynamic epigenetic trajectories of bone cells. Specifically, researchers should leverage advanced single-cell epigenomics and in vivo lineage-tracing technologies to elucidate spatial and temporal epigenetic dynamics across different stages of osteoporosis. Additionally, elucidating the context-specific functions of emerging RNA modifications (such as m6A, ac4C, and m5C) and decoding how they operate within distinct skeletal microenvironments should be prioritized. These avenues will provide a more granular understanding of osteoporosis pathogenesis and help identify precise, novel therapeutic targets.

#### Mechanistic synthesis

While the epigenetic landscape of osteoporosis is complex, a higher-level integration reveals that diverse upstream etiologies (aging, estrogen deficiency, glucocorticoids, and metabolic stress) ultimately converge on shared downstream signaling pathways, primarily the Wnt/β-catenin and Runx2 axes. Notably, another critical shared consequence is osteoblast ferroptosis. Across multiple osteoporosis subtypes, epigenetic dysregulations directly silence key anti-ferroptotic genes (such as Gpx4), thereby triggering this pathological process and accelerating bone loss. Crucially, these epigenetic modifications do not operate in isolation but form intricate, cross-talking networks. For example, non-coding RNAs act as structural scaffolds to recruit histone-modifying enzymes like EZH2, while aberrant DNA methylation can conversely silence the transcription of key non-coding RNAs. Moving forward, overcoming current translational bottlenecks will require shifting from single-mechanism studies to decoding these integrated epigenetic networks, which will be essential for developing comprehensive and robust therapeutic strategies.

## Epigenetic modification-based therapeutics for osteoporosis

**Fig. 3:**
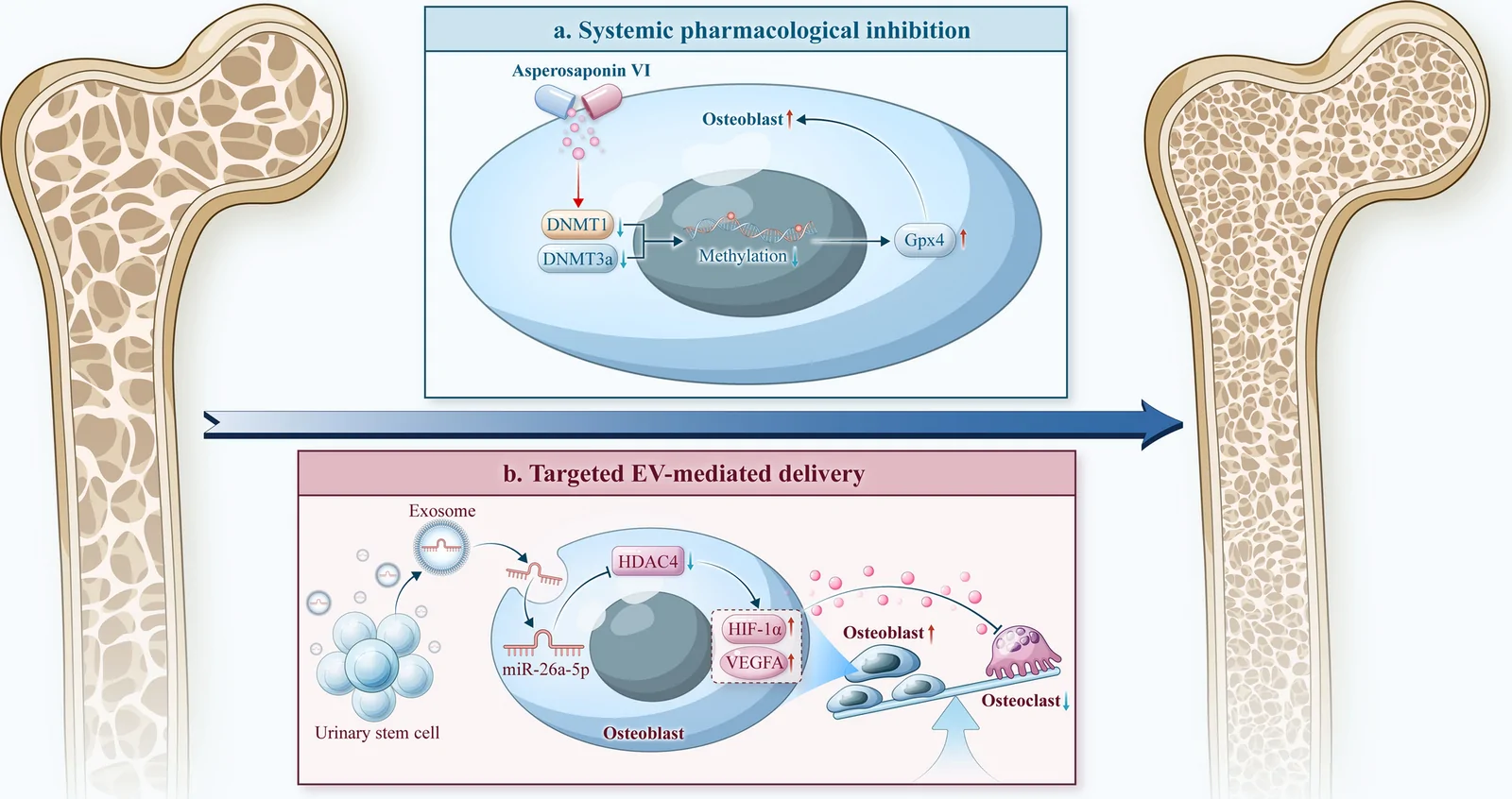
Therapeutic strategies based on epigenetic modifications in Osteoporosis. **a** Systemic pharmacological inhibition: Administration of Asperosaponin VI, a DNMT inhibitor, induces the degradation of DNMT1/DNMT3a, thereby reducing DNA methylation levels. This demethylation restores Gpx4 expression, promoting osteoblast differentiation. **b** Targeted EV-mediated delivery: EVs secreted by urinary stem cells deliver enriched miR-26a-5p to osteogenic precursor cells. Intracellularly, miR-26a-5p targets and degrades HDAC4 transcripts, activating the HIF-1α/VEGFA signaling cascade to enhance osteoblast differentiation and inhibit osteoclast activity

Therapeutic strategies based on epigenetic modifications modulate epigenetic regulatory networks to rebalance osteogenesis, osteoclastogenesis, and inflammatory signaling, thereby inhibiting the onset and progression of osteoporosis, as summarized in Table 2 and Fig. 3. These interventions control stem cell fate, cellular senescence, and immune–bone coupling, ultimately restoring bone homeostasis and improving osteoporotic bone loss.

**Table 2:** Therapeutic strategies based on epigenetic modifications in Osteoporosis

Osteoporosis	Intervention	Targets	Function	Study model	Refs
Senile osteoporosis	Injection of resveratrol and vitamin C	TRIM37/PARP1, TET1, DNMT1	Osteogenesis ↑	In vitro & Animals	[183]
	CXCR4-targeted EVs delivering GSH	TET1, GSS, GSH	Osteogenesis ↑	In vitro & Animals	[17]
	Injection or hydrogel delivery of iBP	PRC1.1, KDM2B, H2AK119ub1, Wnt/β-catenin	Osteogenesis ↑	In vitro & Animals	[184]
	AAV-mediated METTL3 overexpression	METTL3, m6A, Hspa1a, YTHDF2	Osteogenesis ↑	In vitro & Animals	[18]
	Lipid nanoparticles delivering circRNA	MMP9, p21, MMP3	Osteogenesis ↑	In vitro & Animals	[19]
Postmenopausal osteoporosis	Injection of iron chelation, anti-ferroptosis agents, or Nrf2 agonists	Nrf2, DNMT3a, RANKL	Osteoclastogenesis ↓	In vitro & Animals	[185]
	Injection of GW9662	DNMT3a, PPARγ, SCD1, GLUT1	Osteogenesis ↑	In vitro & Animals	[186]
	Injection of α-KG	JMJD7, H3R2me1, H3K27me3, IDH, Runx2, α-KG	Osteogenesis ↑	In vitro & Animals	[187]
	Injection of TC-E 5002	KDM7A, H3K9me2, H3K27me2, Fap, Rank1, Wnt/β-catenin	Osteogenesis ↑ Osteoclastogenesis ↓	In vitro & Animals	[16]
	Injection of EPZ015666	PRMT5, H3R8me2s, H4R3me2s Cxcl10, Rsad2	Osteoclastogenesis ↓	In vitro & Animals	[188]
	Injection of melatonin	PRMT1, NF-κB, NFATc1, TRAP	Osteoclastogenesis ↓	In vitro & Animals	[189]
	Injection of CARM1	PPP1CA, AKT, AMPK, PFK1, PFKFB3	Osteogenesis ↑ Osteoclastogenesis ↓	In vitro & Animals	[190]
	Overexpression of METTL14 or HOXA5	METTL14, m6A, HOXA5, WNK1	Osteoclastogenesis ↓	In vitro & Animals	[191]
	Injection of ISO	YBX1, m5C, BGLAP	Osteogenesis ↑	In vitro & Animals	[192]
	Overexpression of lncRNA KCNQ1OT1	lncRNA KCNQ1OT1, miR-421-3p, mTOR	Osteogenesis ↑	In vitro & Animals	[193]
Glucocorticoid Induced Osteoporosis	Knockdown of DNMT1 or overexpression of lncRNA H19	DNMT1, lncRNA H19, GSK-3β, β-catenin	Osteogenesis ↑	In vitro & Animals	[194]
	Overexpression of lncRNA TERC	lncRNA TERC, EZH2, H3K27me3, DKK1, Wnt/β-catenin	Osteogenesis ↑	In vitro	[195]
	EVs from myoblasts delivering miR-92 a-3p	miR-92 a-3p, PTEN, PI3K/AKT	Osteogenesis ↑	In vitro & Animals	[196]
	Overexpression of lncRNA TRG-AS1	lncRNA TRG-AS1, miR-802, CAB39, AMPK/SIRT-1, NF-κB	Osteogenesis ↑	In vitro & Animals	[197]
	Overexpression of circRNA_0006393	circRNA_0006393, miR-145-5p, FOXO1, RUNX2, BMP2	Osteogenesis ↑	In vitro & Animals	[198]
Diabetic osteoporosis	Injection of AVI	DNMT1, DNMT3a, Gpx4	Osteogenesis ↑	In vitro & Animals	[15]
	Implantation of DNMT3a-silenced ADSCs	DNMT3a, Wnt/β-catenin	Osteogenesis ↑	In vitro & Animals	[150]
	Injection of Probiotics or indole −3-propionic acid	TLR4, miR-138, JMJD3, H3K27me3, Tfam	Osteogenesis ↑	In vitro & Animals	[199]
	Injection of EVs from urine-derived stem cells delivering miR-26 a-5p	miR-26 a-5p, HDAC4, HIF-1α/VEGFA	Osteogenesis ↑ Osteoclastogenesis ↓	In vitro & Animals	[200]
	Injection of phytic acid	circEIF4B, miR-186-5p, FOXO1, IGF2BP3, ITGA5	Osteogenesis ↑	In vitro & Animals	[201]

↑ indicates upregulation or promotion

↓ indicates downregulation or inhibition

### Therapeutics for Senile osteoporosis

Aberrant alterations in DNA methylation are fundamental drivers of BMSC senescence and bone loss in senile osteoporosis, making targeted DNA methylation a promising therapeutic strategy. Under normal conditions, the TRIM37/PARP1 complex recruits TET1 to catalyze the demethylation of DNMT1 exons. This targeted demethylation facilitates CTCF binding, which is essential to prevent aberrant alternative splicing and ensure the expression of full-length DNMT1. However, during aging, the TRIM37-PARP1-TET1 axis is impaired, resulting in the aberrant alternative splicing and depletion of full-length DNMT1, which ultimately inhibits the osteogenic differentiation of BMSCs. Consequently, dual pharmacological activation using resveratrol (an ATM activator) and vitamin C (a TET activator) can reactivate this axis, rejuvenating aged BMSCs and mitigating osteoporotic bone loss in vivo in mouse models [183]. Furthermore, the decline of TET1 also disrupts antioxidant defense mechanisms. In aged mice, the age-related decline of TET1 leads to the hypermethylation of the glutathione synthetase (GSS) promoter, thereby downregulating GSS expression and inhibiting endogenous glutathione (GSH) synthesis. This cripples the antioxidant defense system and suppresses the osteogenic potential of BMSCs. To rescue this metabolic defect, a CXCR4-targeted EV platform was developed to deliver GSH into the bone marrow niche. Ultimately, in vivo validation in aged mice demonstrated that this intervention suppresses oxidative stress, improves mitochondrial function, delays cellular senescence, and reduces bone loss in aged bone tissue [17]. Collectively, these findings underscore that rectifying age-induced DNA methylation imbalances holds immense translational potential for rejuvenating the aged bone microenvironment.

Therapeutic strategies directed at aberrant histone modifications represent another critical frontier in combating senile osteoporosis. During skeletal aging, the Polycomb repressive complex 1.1 (PRC1.1), guided to chromatin by KDM2B, promotes H2AK119ub1 levels. This pathological accumulation represses the expression of Wnt/β-catenin signaling components and osteogenic transcription factors, elevating the transcriptional activation threshold for osteogenic signals and thereby inhibiting osteogenic differentiation. iBP, a small-molecule inhibitor, suppresses PRC1.1 assembly to reduce H2AK119ub1 levels, thereby restoring the expression of the Wnt/β-catenin pathway and promoting osteogenic differentiation. Both systemic administration and local hydrogel-mediated delivery of iBP accelerate bone repair and rescue osteoporotic bone deterioration in vivo in senile mouse models [184].

In senile osteoporosis, the age-dependent depletion of METTL3 drives osteoblast senescence and compromises bone formation. To counteract this aging phenotype, overexpressing METTL3 in osteoblasts via adeno-associated virus (AAV) restores the m6A methylation of Hspa1a mRNA and recruits the m6A reader YTHDF2. Notably, while the general function of YTHDF2 is to promote mRNA degradation, in this context it promotes the stability of Hspa1a transcripts. The resulting accumulation of Hspa1a suppresses senescence-associated regulators, rejuvenates osteoblast activity, and ameliorates bone loss in vivo in aging mice [18].

The targeted delivery of non-coding RNAs provides an innovative approach to reprogram the senescence-associated microenvironment. In the context of senile osteoporosis, elevated levels of MMP9 within the skeletal microenvironment drive BMSC senescence and disrupt the osteoblast-osteoclast balance. To combat this age-related skeletal degeneration, a novel in vivo circRNA engineering strategy has been developed utilizing phosphatidylserine-modified lipid nanoparticles. Subsequent experiments in mouse models verified that these biomimetic nanoparticles promote monocytes/macrophages to locally secrete anti-MMP9 neutralizing antibodies within the bone niche. This suppresses the senescence-associated secretory phenotype, downregulates senescence markers such as p21 and MMP3, and ultimately restores the osteogenic capacity of aged BMSCs [19].

Collectively, these emerging therapies dismantle the aging-driven microenvironment by rectifying aberrant epigenetic modifications. Ultimately, this reverses cellular senescence, rejuvenates the skeletal niche, and accelerates bone regeneration in the elderly.

### Therapeutics for postmenopausal osteoporosis

Estrogen deficiency induces pathological iron accumulation, leading to lipid peroxidation and subsequent osteocyte ferroptosis. This ferroptotic stress suppresses the Nrf2 pathway and subsequently inhibits Dnmt3a, thereby decreasing the methylation level of the RANKL promoter. This leads to the upregulation of RANKL expression, which overactivates osteoclastogenesis. Pharmacological interventions targeting this upstream mechanism, such as iron chelation, anti-ferroptosis agents, or Nrf2 agonists, can restore DNMT3a expression, re-establish RANKL promoter methylation, and protect against osteoclast-mediated bone deterioration in vivo in mouse models [185]. Beyond its essential role in restraining osteoclastogenesis, DNMT3a is equally vital for osteoblast differentiation. In PMOP, DNMT3a decreases, reducing the methylation of the peroxisome proliferator-activated receptor γ (PPARγ) promoter and promoting PPARγ expression. Subsequently, the upregulated stearoyl-coenzyme A desaturase 1 (SCD1) inhibits osteoblast differentiation by downregulating the glucose transporter GLUT1. To counteract this metabolic dysfunction, utilizing the specific PPARγ inhibitor GW9662 restores GLUT1-dependent energy metabolism, rejuvenates osteoblast differentiation, and alleviates osteoporotic bone loss in vivo in mice [186]. Collectively, these dual mechanisms reveal that restoring Dnmt3a-mediated methylation represents a comprehensive strategy for rebalancing bone remodeling in PMOP.

The dynamic remodeling of histone methylation plays an equally pivotal role in the pathogenesis of PMOP. Under estrogen deficiency, the depletion of the histone arginine demethylase JMJD7 triggers the abnormal accumulation of repressive H3R2me1 and H3K27me3 marks at the promoters of IDH and the master osteogenic transcription factor Runx2. The resulting deficiency of IDH sharply reduces α-KG synthesis, forcing osteoblasts into metabolic dysfunction. Concurrently, the depletion of RUNX2 halts osteogenic differentiation. Supplementing with the tricarboxylic acid cycle intermediate α-KG bypasses this metabolic blockade, erasing the repressive methylation marks, stabilizing the RUNX2 protein, and ultimately rescuing osteoporotic bone loss in vivo in OVX mice [187]. Conversely, while the loss of JMJD7 impairs osteogenesis, the unabated activity of histone lysine demethylase KDM7A exacerbates bone deterioration by reciprocally skewing the osteoblast-osteoclast axis. In osteoprogenitors, KDM7A removes the repressive di-methylation marks H3K9me2 and H3K27me2 from the promoters of Fap and Rankl. The consequent upregulation of FAP inactivates the Wnt/β-catenin signaling to halt osteoblast differentiation, whereas upregulation of RANKL stimulates osteoclastogenesis. Targeted inhibition of KDM7A, either via genetic deletion or selective pharmacological inhibitors like TC-E 5002, restores the hypermethylated state of the Fap and Rankl promoters. This not only promotes Wnt-driven osteogenic differentiation but also inhibits osteoclast differentiation, thereby preventing OVX-induced osteoporosis in vivo [16]. Parallel to demethylases, inhibiting specific histone methyltransferases also attenuates pathological bone resorption. Pharmacological blockade of protein arginine methyltransferase 5 (PRMT5) using the selective inhibitor EPZ015666 significantly decreases the enrichment of H3R8me2s and H4R3me2s marks at the promoters of Cxcl10 and Rsad2. This epigenetic modulation directly suppresses the expression levels of Cxcl10 and Rsad2, ultimately inhibiting osteoclast differentiation and mitigating ovariectomy-induced postmenopausal osteoporosis in vivo [188]. Similarly, targeting PRMT1 offers another effective approach to restrict osteoclast activity. Via a melatonin receptor-independent pathway, melatonin inhibits the PRMT1-mediated signaling cascade, thereby interfering with the binding of PRMT1 to the NF-κB subunit p65 and suppressing its transcriptional activity. This blockade downregulates the expression of osteoclast-specific genes such as NFATc1 and TRAP, ultimately mitigating ovariectomy-induced bone loss in vivo [189]. Beyond regulating transcription factors and histones, arginine methylation also governs bone homeostasis by reprogramming cellular energy metabolism. CARM1 shifts the metabolic profile of the skeletal microenvironment from oxidative phosphorylation (OXPHOS) to aerobic glycolysis. Mechanistically, in vivo supplementation of CARM1 promotes the methylation of the R23 residue of PPP1CA and restricts its dephosphorylation activity, thereby promoting the phosphorylation and activation of AKT and AMPK. This subsequently increases the activity of PFK1 and PFKFB3, improving glycolytic flux. Concurrently, CARM1 upregulates the expression of PDK3 to inhibit the tricarboxylic acid cycle and restrict mitochondrial OXPHOS. Because osteoclast differentiation depends on OXPHOS whereas osteoblastogenesis favors aerobic glycolysis, this CARM1-mediated metabolic reprogramming strongly promotes osteogenic differentiation while impairing osteoclast formation, ultimately mitigating ovariectomy-induced bone loss [190]. Collectively, these findings demonstrate that manipulating specific histone modifiers provides a precise therapeutic strategy for PMOP.

Targeting mRNA methylation also presents a crucial therapeutic strategy for mitigating PMOP. In the estrogen-deficient bone microenvironment, the downregulation of METTL14 suppresses the m6A methylation of HOXA5, thereby decreasing its mRNA stability and downregulating HOXA5 expression. This depletion subsequently downregulates WNK1, unleashing NLRP3-dependent macrophage pyroptosis and driving osteoclast differentiation. Targeted overexpression of METTL14 or HOXA5 restores WNK1 transcription, blocks macrophage pyroptosis, and alleviates osteoporotic bone loss in vivo in OVX mice [191]. Similarly, targeting m5C modification is another promising therapeutic strategy. Administration of isoimperatorin (ISO) to OVX mice elevates YBX1 expression, enhancing the m5C modification of BGLAP mRNA, thereby increasing its stability and expression. Ultimately, ISO administration ameliorates trabecular bone loss and rescues the osteoporotic phenotype in vivo [192].

Modulating complex non-coding RNA networks represents another crucial aspect of therapy. In PMOP, estrogen deficiency downregulates the expression of the lncRNA KCNQ1OT1, impairing its capacity to sequester miR-421-3p. The resulting elevation of miR-421-3p represses mTOR expression, which subsequently inhibits the expression of key osteogenic factors, ultimately attenuating osteoblast activity. Overexpressing KCNQ1OT1 reverses this process, reconstructing the KCNQ1OT1/miR-421-3p/mTOR regulatory axis, restoring osteoblast activity, and ameliorating bone deterioration in mouse models [193].

In summary, these multifaceted epigenetic interventions effectively counteract estrogen deficiency-induced bone deterioration, providing a precise and highly promising approach to restoring the osteoblast-osteoclast balance and rejuvenating bone remodeling in postmenopausal women.

### Therapeutics for glucocorticoid-induced osteoporosis

In glucocorticoid-induced skeletal disorders, dysregulated DNA methylation acts as a critical epigenetic catalyst that diverts BMSC lineage commitment from osteogenesis toward adipogenesis. Rectifying these aberrant methylation profiles offers a powerful therapeutic avenue. In glucocorticoid-induced osteonecrosis of the femoral head, DNMT1 hypermethylates the promoter of the lncRNA H19, leading to its downregulated expression. The depletion of lncRNA H19 activates GSK-3β to promote β-catenin degradation, which stimulates the adipogenic differentiation of BMSCs and suppresses their osteogenic differentiation. Knockdown of DNMT1 or overexpression of H19 restores β-catenin stability. Ultimately, in vivo studies in rat models demonstrated that these epigenetically corrected BMSCs exhibit restored osteogenic capacity and reduced adipogenesis, mitigating glucocorticoid-induced osteonecrosis [194].

Beyond DNA methylation, the post-transcriptional regulatory network orchestrated by non-coding RNAs adds another perspective to the pathogenesis and therapeutics of GIOP. For instance, in BMSCs of GIOP, overexpressing the lncRNA TERC promotes its interaction with EZH2 and recruits EZH2 to the DKK1 promoter, thereby elevating H3K27me3 levels and suppressing DKK1 expression. The suppression of DKK1 restores the activity of the Wnt/β-catenin pathway, ultimately recovering the osteogenic capacity of BMSCs and reversing glucocorticoid-induced cellular suppression in vitro [195]. In addition to intracellular lncRNA pathways, intercellular epigenetic communication via exosomal microRNAs offers a novel therapeutic paradigm. Specifically, exosomes secreted by mechanical stress-stimulated myoblasts are enriched with miR-92 a-3p. Upon internalization by BMSCs, this microRNA targets and suppresses PTEN transcripts, thereby robustly activating the osteogenic PI3K/AKT signaling cascade. This rescues BMSCs from glucocorticoid-induced suppression, restores their osteogenic differentiation, and ultimately ameliorates trabecular bone deterioration in vivo in GIOP mouse models [196]. Furthermore, in GIOP, overexpression of the lncRNA TRG-AS1 relieves the repression of its downstream target CAB39 via competitive binding to miR-802. The subsequent accumulation of CAB39 activates the osteoprotective AMPK/SIRT-1 signaling cascade while simultaneously suppressing NF-κB activity. Ultimately, this restores osteoblast proliferation and differentiation and ameliorates trabecular bone deterioration in vivo in rat models [197]. Similarly, circRNAs participate in this intricate crosstalk to protect osteogenic targets. Overexpressed circRNA_0006393 acts as a molecular sponge to sequester miR-145-5p, thereby relieving the repression of its downstream target FOXO1. The accumulation of FOXO1 enhances the expression of core osteogenic markers, including RUNX2 and BMP2. Ultimately, reconstructing this circRNA/miRNA/mRNA regulatory axis restores the osteogenic capacity of BMSCs and significantly ameliorates trabecular bone deterioration in vivo in mice [198].

Collectively, these findings highlight the critical role of epigenetic dysregulation in the pathogenesis of GIOP. Targeted interventions hold immense clinical potential for reversing the osteo-adipogenic imbalance and ameliorating glucocorticoid-induced skeletal deterioration.

### Therapeutics for diabetic osteoporosis

In DOP, the pathological hyperglycemic and lipotoxic environment aberrantly upregulates DNMTs, inhibiting osteoblast activity and the osteogenic potential of BMSCs. Specifically, DOP triggers an elevation of DNMT1 and DNMT3a, which hypermethylate the promoter of Gpx4. This leads to decreased Gpx4 expression, triggering lipid peroxidation and subsequent osteoblast ferroptosis, exacerbating diabetic bone loss. To combat this, Asperosaponin VI (AVI), a potent natural DNMT inhibitor, promotes the lysosomal degradation of DNMT1/3 a. Consequently, AVI treatment demethylates the Gpx4 promoter, restores Gpx4 expression, and protects osteoblasts from ferroptotic death in vivo in diabetic mice (Fig. 3a) [15]. Beyond inducing osteoblast ferroptosis, aberrant DNMT3a upregulation sabotages the bone-regenerative potential of ADSCs in DOP. The DOP-induced accumulation of DNMT3a methylates genomic targets, which suppresses the Wnt/β-catenin pathway and halts osteogenic differentiation. Targeted silencing of DNMT3a using shRNA decreases this pathological DNA methylation, reactivates the Wnt/β-catenin cascade, and rescues the osteogenic capacity of ADSCs. Furthermore, implanting these DNMT3a-silenced ADSCs enhances new bone formation and defect repair in vivo in diabetic mouse models [150]. Collectively, these findings highlight that dismantling the DNMT-driven hypermethylation network represents a promising and targeted strategy for ameliorating DOP.

Beyond targeting DNA methylation, therapeutic interventions directed at non-coding RNA and histone modification networks also hold great promise for ameliorating DOP. Obesity-driven metabolic endotoxemia hyperactivates TLR4 signaling in osteoblasts, which upregulates miR-138. This microRNA suppresses the histone demethylase JMJD3 (KDM6B), leading to an accumulation of the repressive H3K27me3 mark at the promoter of Tfam. This decreases Tfam expression, inducing aberrant energy metabolism in osteoblasts, ultimately inhibiting osteogenic differentiation. Oral supplementation with probiotics or the specific gut microbiota-derived metabolite indole −3-propionic acid suppresses TLR4 activation in osteoblasts, thereby downregulating miR-138 and restoring JMJD3 demethylase activity. Consequently, this restored activity ultimately revives Tfam-dependent mitochondrial respiration and rescues osteogenic differentiation in vivo in diabetic mice [199].

Utilizing EVs can reprogram the epigenetic landscape in DOP. EVs secreted by urine-derived stem cells are enriched with miR-26 a-5p. Upon isolation and injection into DOP rats, these EVs are internalized by osteogenic precursor cells, where this microRNA targets and represses HDAC4 transcripts. This targeted repression unleashes the HIF-1α/VEGFA signaling cascade, ultimately enhancing osteoblast differentiation, inhibiting osteoclast activity, and ameliorating trabecular bone deterioration in vivo in DOP rats (Fig. 3b) [200]. Concurrently, under hyperglycemic microenvironments, metabolic interventions can rejuvenate BMSC function by orchestrating complex circRNA networks. For instance, treatment with the natural compound phytic acid upregulates circEIF4B expression in BMSCs. As a molecular sponge, circEIF4B sequesters miR-186-5p to relieve its repression of the osteogenic transcription factor FOXO1. Simultaneously, circEIF4B binds to IGF2BP3 to prevent its ubiquitin-mediated degradation. The stabilized IGF2BP3 subsequently binds and enhances the mRNA stability of ITGA5. Ultimately, reconstructing this dual regulatory axis restores the osteogenic capacity of BMSCs and alleviates diabetic bone loss in vivo in rat models [201].

Collectively, these emerging therapeutic strategies underscore the immense clinical potential of epigenetic interventions in DOP. By rectifying aberrant DNA methylation, histone modifications, and non-coding RNA networks, these interventions can reverse diabetes-induced osteogenic suppression and accelerate bone regeneration.

### Summary of therapeutics and limitations

The aforementioned studies highlight the immense potential of epigenetic interventions across various osteoporosis subtypes. By rectifying aberrant DNA methylation, dynamic histone modifications, mRNA modifications, and complex non-coding RNA networks, these strategies rebalance the osteoblast-osteoclast axis, restoring the osteogenic activity of BMSCs or suppressing the aberrant activation of osteoclasts.

**Fig. 4:**
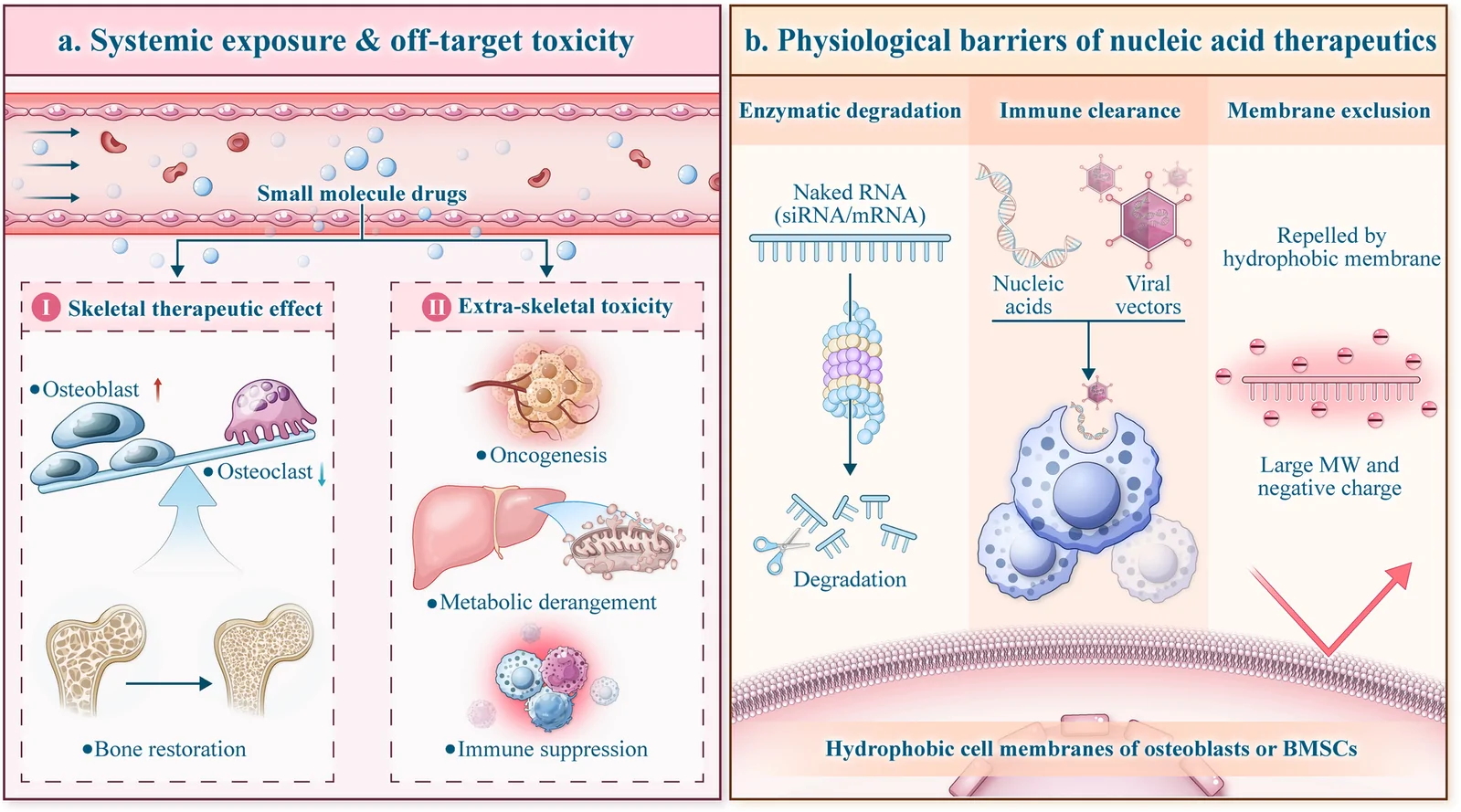
Current limitations of skeletal pharmacotherapy: systemic toxicity and nucleic acid delivery barriers. **a** Systemic exposure & off-target toxicity: Beyond their intended skeletal pharmacological effects, conventional small molecule drugs often suffer from non-specific systemic exposure, leading to severe extra-skeletal toxicities such as oncogenesis and immune suppression. **b** Physiological barriers of nucleic acid therapeutics: Emerging nucleic acid therapeutics offer high specificity but face formidable physiological barriers. These barriers include rapid enzymatic degradation, immune system clearance, and the inability to penetrate target cells due to electrostatic repulsion from hydrophobic cell membranes

Among the aforementioned therapeutic strategies, many rely on broad-spectrum epigenetic inhibitors or systemically administered pharmacological agents. However, most epigenetic regulators (such as DNMTs, HDACs, and m6A methyltransferases) are ubiquitously expressed and govern fundamental cellular processes across all organ systems. While these agents may modulate epigenetic modifications to restore the expression of target genes like Gpx4 or Runx2 in the bone marrow to rescue osteogenesis, they simultaneously disrupt the epigenetic landscape in non-skeletal tissues. This non-specific modulation can pose severe off-target effects and safety concerns, including potential oncogenesis, immune suppression, or metabolic derangement (Fig. 4a). Therefore, systemic modulation of epigenetic regulators easily leads to unacceptable toxicities, restricting their long-term clinical application in osteoporosis.

To circumvent these systemic toxicities, the field has begun adopting advanced delivery platforms, such as engineered EVs, adeno-associated viruses, and lipid nanoparticles, for the targeted modulation of epigenetic modifications in preclinical models. By concentrating epigenetic modulators exclusively within the skeletal niche, these targeted interventions rebalance bone remodeling while minimizing peripheral exposure, offering a safer and comprehensive approach for restoring bone homeostasis.

Despite these successful preclinical results, developing viable clinical therapies still faces substantial obstacles. The difficulty of drug delivery remains a major hurdle for their clinical application. A vast majority of these emerging therapies rely on manipulating non-coding RNAs or employing viral vectors. The clinical application of these nucleic acid-based epigenetic modulators faces physiological barriers (Fig. 4b). First, naked RNA molecules are unstable and susceptible to rapid degradation by endogenous RNases in the systemic circulation. Second, their negative charge and large molecular weight prevent them from passively crossing the hydrophobic cell membranes of osteoblasts or BMSCs. Third, the systemic administration of exogenous nucleic acids or viral vectors often triggers robust immunogenic responses, leading to their rapid clearance before reaching the skeletal microenvironment.

In summary, the future of epigenetic therapeutics in osteoporosis lies not only in discovering new targets but, more crucially, in innovating tissue-specific delivery platforms. Only by achieving spatial precision, editing the epigenome strictly within the bone microenvironment, can we overcome current safety and delivery bottlenecks, unlocking the true clinical value of epigenetic therapeutics.

## Conclusions and future perspectives

Epigenetic modifications, encompassing DNA methylation, histone modifications, mRNA modifications, and non-coding RNAs, are fundamental drivers across diverse osteoporosis subtypes. Summarizing these regulatory networks helps reveal the shared and unique drivers of bone loss, providing a comprehensive theoretical foundation for targeted interventions. At the cellular level, these modifications dictate bone homeostasis by modulating the gene networks that govern osteoblast and osteoclast fate and function. Consequently, their aberrant regulation disrupts the bone remodeling balance and drives osteoporotic progression. Importantly, the reversible nature of these epigenetic changes offers promising opportunities for therapeutic intervention. Preclinical evidence supports the feasibility of reprogramming aberrant epigenetic states to rebalance bone remodeling and halt osteoporosis progression.

While this review comprehensively maps the epigenetic landscape and therapeutic potential in osteoporosis, it is important to acknowledge certain inherent limitations of our study. First, as a narrative literature review rather than a systematic review or meta-analysis, our literature selection process lacked rigidly defined search protocols and quantitative bias assessment tools. This methodology carries a potential risk of selection bias, as it may inadvertently emphasize studies reporting positive preclinical outcomes. Second, to provide a broad and comprehensive overview encompassing multiple diverse osteoporosis subtypes (such as senile, postmenopausal, and diabetic osteoporosis), we had to synthesize the literature at a macroscopic level, which limited our in-depth exploration of the specific molecular mechanisms for each subtype. Finally, because the field of epigenetics is advancing so rapidly, new mechanisms and therapeutic targets are continuously being discovered. Therefore, this review may not capture the very latest ongoing studies that emerged during the publication process.

To comprehensively decode these regulatory networks, future investigations must transition to spatial epigenomic and multi-omics approaches. Integrating spatial epigenomics with transcriptomics, proteomics, and metabolomics will enable the systematic identification of interconnected pathogenic feedback loops underlying bone loss.

Furthermore, the comprehensive molecular signatures identified through these multi-omics approaches will drive biomarker discovery. Circulating epigenetic signatures hold immense promise as non-invasive biomarkers for early detection and prognostic validation. However, transitioning them to the clinic demands large-scale, multicenter trials and standardized detection protocols. Establishing these reliable biomarkers will subsequently enable precision epigenetic interventions tailored to subtype-specific osteoporosis.

Therapeutically, exploring novel combinatorial strategies represents a critical future direction. Integrating highly specific epigenetic modulators with conventional anti-osteoporotic agents could unlock potent synergistic effects, circumventing potential drug resistance and maximizing bone regeneration. Finally, advancing targeted delivery platforms from preclinical models into large animal models is an indispensable step to rigorously validate their pharmacokinetics and long-term safety profiles. Ultimately, beyond biological efficacy, establishing scalable manufacturing protocols and evaluating cost-effectiveness will be decisive in translating these preclinical discoveries into accessible clinical cures.

## Data Availability

Not applicable. No new data were created or analyzed in this study.

## Abbreviations

PMOP: Postmenopausal osteoporosis
GIOP: Glucocorticoid-induced osteoporosis
DNMT: DNA methyltransferase
5-mC: 5-Methylcytosine
CTCF: CCCTC-binding factor
TET: Ten–eleven translocation
E1: Ubiquitin-activating enzymes
E2: Ubiquitin-conjugating enzymes
E3: Ubiquitin ligases
m6A: N6-methyladenosine
ac4C: N4-acetylcytosine
m5C: 5-Methylcytosine
NAT10: N-acetyltransferase 10
lncRNA: Long non-coding RNA
miRNA: MicroRNA
circRNA: Circular RNA
Gal-1: Galectin-1
MeCP2: Ethyl-CpG binding protein 2
α-KG: α-Ketoglutarate
NAP1L2: Nucleosome assembly protein 1‐like 2
CIC: Citrate carrier
OVX: Ovariectomized
KLF5: Krüppel-like factor 5
BMDMs: Bone marrow-derived macrophages
SFRP1: Secreted frizzled related protein 1
OPG: Osteoprotegerin
CEBPA: C/ebp α
ACE: Angiotensin converting enzyme
RAS: Renin-angiotensin system
PDE: Prenatal dexamethasone exposure
CORT: Corticosterone
GRα: Glucocorticoid receptor α
IGF1: Insulin-like growth factor 1
11β-HSD2: 11-β Hydroxysteroid dehydrogenase 2
DOP: Diabetic osteoporosis
AGEs: Advanced glycation end products
CALCA: Calcitonin gene-related peptide alpha
OPN: Osteopontin
HFD: High-fat diet
SMG: Simulated microgravity
BCa: Breast cancer
MM: Multiple myeloma
LPS: Lipopolysaccharide
GSS: Glutathione synthetase
GSH: Glutathione
PRC1.1: Polycomb repressive complex 1.1
AAV: Adeno-associated virus
PPARγ: Peroxisome proliferator-activated receptor γ
SCD1: Stearoyl-coenzyme A desaturase 1
PRMT5: Protein arginine methyltransferase 5
ISO: Isoimperatorin
AVI: Asperosaponin VI
